# Angioembolization in patients with blunt splenic trauma in Germany –guidelines vs. Reality a retrospective registry-based cohort study of the TraumaRegister DGU®

**DOI:** 10.1007/s00068-024-02640-6

**Published:** 2024-09-16

**Authors:** Benny Kölbel, Sebastian Imach, Michael Engelhardt, Arasch Wafaisade, Rolf Lefering, Christian Beltzer, TraumaRegister DGU

**Affiliations:** 1 Department of General, Visceral and Thoracic Surgery, German Armed Forces Hospital Ulm, Oberer Eselsberg 40, 89081 Ulm, Germany; 2 Department of Trauma and Orthopedic Surgery, Cologne-Merheim Medical Center (CMMC), University Witten/Herdecke, Cologne, Germany; 3 Department of Vascular and Endovascular Surgery, German Armed Forces Hospital Ulm, Ulm, Germany; 4 Institute for Research in Operative Medicine, Witten/Herdecke University, Cologne, Germany; 5 Committee On Emergency Medicine, Intense Care and Trauma Management (Sektion NIS) of the German Trauma Society (DGU), Berlin, Germany

**Keywords:** Blunt splenic injury, Trauma, Angioembolization, Splenectomy, WSES Spleen Trauma Management Algorithm

## Abstract

**Purpose:**

Nonoperative management (NOM) for blunt splenic injuries (BSIs) is supported by both international and national guidelines in Germany, with high success rates even for severe organ injuries. Angioembolization (ANGIO) has been recommended for stabilizable patients with BSI requiring intervention since the 2016 German National Trauma Guideline. The objectives were to study treatment modalities in the adult BSI population according to different severity parameters including NOM, ANGIO and splenectomy in Germany.

**Methods:**

Between 2015 and 2020, a retrospective registry-based cohort study was performed on patients with BSIs with an Abbreviated Injury Score ≥ 2 in Germany using registry data from the TraumaRegister DGU® (TR DGU). This registry includes patients which were treated in a resuscitation room and spend more than 24-h in an intensive care unit or died in the resuscitation room.

**Results:**

A total of 2,782 patients with BSIs were included in the analysis. ANGIO was used in 28 patients (1.0%). NOM was performed in 57.5% of all patients, predominantly those with less severe organ injuries measured by the American Association for the Surgery of Trauma Organ Injury Scale (AAST) ≤ 2. The splenectomy rate for patients with an AAST ≥ 3 was 58.5%, and the overall mortality associated with BSI was 15%.

**Conclusions:**

In this cohort splenic injuries AAST ≥ 3 were predominantly managed surgically and ANGIO was rarely used to augment NOM. Therefore, clinical reality deviates from guideline recommendations regarding the use of ANGIO and NOM. Local interdisciplinary treatment protocols might close that gap in the future.

**Supplementary Information:**

The online version contains supplementary material available at 10.1007/s00068-024-02640-6.

## Introduction

In blunt abdominal and thoracic trauma, the spleen is one of the most commonly injured organs [1–3]. The nonoperative management (NOM) of blunt splenic injuries (BSI) has become the worldwide evidence-based standard of care for hemodynamically stable patients, with high success rates, even for severe organ injuries [4–7]. It is therefore supported by both international and German national guidelines [8–11].

Angioembolization (ANGIO) was shown to be effective in selected patients as an adjunct to NOM. Factors associated with an increased efficacy of ANGIO are still subject of discussion, but commonly high-grade splenic injuries > 3 AAST and the presence of signs of vascular injuries are accepted as criteria for ANGIO [12]. ANGIO improved spleen salvage rates and reduced mortality in several studies [4, 5, 13–15]. ANGIO has been recommended since 2016 in the German National Trauma Guideline. The guideline recommendation can be translated as follows: “In case of splenic injuries requiring intervention, selective angioembolization should be performed instead of operative management in hemodynamically stabilizable patients.” Grade of recommendation (GoR) B [10].

Splenic injuries are anatomically classified into five degrees of severity according to the American Association for the Surgery of Trauma Organ Injury Scale (AAST OIS) and Abbreviated Injury Scale (AIS; Table 1) [16]. A clinically relevant extension is the Spleen Trauma Classification by the World Society of Emergency Surgery (WSES; Table 2) [17].

**Table 1:** Splenic Injury Scale (2018 revision)

Anatomical Splenic Injury Scales		
AAST OIS Grade	AIS Severity	Imaging Criteria (based on CT findings)
I	2	Subcapsular hematoma < 10% surface area
		Parenchymal laceration < 1 cm depth Capsular tear
II	2	Subcapsular hematoma 10–50% surface area Intraparenchymal hematoma < 5 cm in diameter
		Laceration 1—3 cm in depth
III	3	Subcapsular hematoma > 50% surface area Ruptured subcapsular or parenchymal hematoma ≥ 5 cm
		Laceration > 3 cm depth
IV	4	Any injury in the presence of a splenic vascular injury* or active bleeding confined within splenic capsule Parenchymal laceration involving segmental or hilar vessels producing > 25% devascularisation
V	5	Shattered spleen Any injury in the presence of splenic vascular injury* with active bleeding** extending beyond the spleen into the peritoneum

*AAST OIS* American Association for the Surgery of Trauma Organ Injury Scale; *AIS* Abbreviated Injury Score

^*^Vascular injury (i.e., pseudoaneurysm or AV fistula) appears as a focal collection of vascular contrast that decreases in attenuation on delayed images

^**^Active bleeding from a vascular injury presents as vascular contrast, focal or diffuse, that increases in size or attenuation in the delayed phase; advance one grade for multiple injuries, each up to grade III[16]

**Table 2:** WSES spleen trauma classification

WSES Spleen Trauma Classification		
WSES class	AAST Grade	Hemodynamic
**MINOR**		
WSES I	I—II	Stable
**MODERATE**		
WSES II	III	Stable
WSES III	IV—V	Stable
**SEVERE**		
WSES IV	I—V	Unstable

*WSES* World Society of Emergency Surgery, *AAST* American Association for the Surgery of Trauma

Treatment options for BSI include NOM, NOM with augmentation by primary ANGIO, NOM with augmentation by secondary ANGIO as a salvage procedure, and early or delayed OM. While spleen-preserving procedures such as partial splenectomy, splenorrhaphy and the application of hemostatic agents are recommended for unstable patients with BSI grades of AAST I–III, splenectomy is recommended for unstable patients with BSI grades of AAST IV–V according to the German guidelines [10].

Laparoscopic surgery for blunt abdominal trauma has been used more frequently in recent years, but splenic injuries in unstable patients are nearly exclusively managed by open surgery [18]. We reviewed research articles on laparoscopic surgery for hemodynamically stable patients with splenic trauma and found similar success rates between open and laparoscopic splenectomy performed by experienced surgeons, but this data cannot be generalized to a nationwide trauma population [19].

The failure rates of NOM (FNOM) with or without ANGIO ranged between 7.7 and 17.4% according to different meta-analyses on the efficacy of NOM for BSI [20–22].

Multiple risk factors for FNOM have been identified, especially high-grade injuries and signs of vascular injury at the initial computer tomography (CT) scan [20, 23]. A “high-grade” BSI is defined as AAST III–V or IV–V according to different publications, with a trend toward recommending prophylactic use of ANGIO in those patients, regardless of the presence of vascular injury and/or active bleeding [4, 24–27].

Signs of splenic vascular injury, such as contrast blush on initial CT, pseudoaneurysms or massive hemoperitoneum, are considered risk factors for failure of NOM. Additionally, depending on the severity of splenic injury, selective ANGIO is recommended for augmenting this strategy [9, 11, 25, 28–30]. The literature shows that the vascular injuries observed on CT might not be reproducible in subsequent ANGIO [31, 32].

The primary purpose of the study was to evaluate the frequency of ANGIO utilization in conjunction with BSI in Germany after its adoption in the 2016 National Trauma Guideline and it related outcomes. Other objectives were the analysis of the following:

- Therapeutic modality rates of NOM vs. OM vs. ANGIO according to the AAST-OIS and the WSES Spleen Trauma Classification (WSES STC)
- Rate of splenectomy according to the AAST-OIS
- Utilization and permeation of ANGIO according to the level of care at the treating trauma center

## Materials and methods

### TraumaRegister DGU®

TR-DGU was founded in 1993. The purpose of this database is to use pseudonymized and standardized documentation of severely injured patients. It is one of the largest trauma registries worldwide with respect to patient numbers, items and data completeness.

The data were collected prospectively in four consecutive time phases from the site of the accident until discharge from the hospital: (A) prehospital phase, (B) emergency room and initial surgery, (C) intensive care unit (ICU) and (D) discharge. The documentation included detailed information on demographics, injury patterns, comorbidities, pre- and in-hospital management, duration in the intensive care unit, and relevant laboratory findings, including transfusion and outcome data for each individual patient. The inclusion criterion was admission to the hospital via the emergency room with subsequent ICU or ICM care or admission to the hospital with vital signs and death before admission to the ICU. The infrastructure for documentation, data management, and data analysis was provided by the Academy for Trauma Surgery GmbH, a company affiliated with the German Trauma Society. The scientific leadership was provided by the Committee on Emergency Medicine, Intensive Care and Trauma Management of the German Trauma Society. The participating hospitals submit their data pseudonymized into a central database via a web-based application. Scientific data analysis was approved according to a peer review procedure laid down in the publication guidelines of TR DGU. Participation is voluntary. For hospitals associated with TraumaNetzwerk DGU®, however, the entry of at least a basic dataset is obligatory for reasons of quality assurance. The TR DGU standard data entry form contains more than 200 items on the severity of injuries and therapies. Data collection is organized by each hospital via an online platform. More than 150 plausibility checks were implemented in the data collection software, including a help function for finding the correct AIS codes.

**Fig.1:**
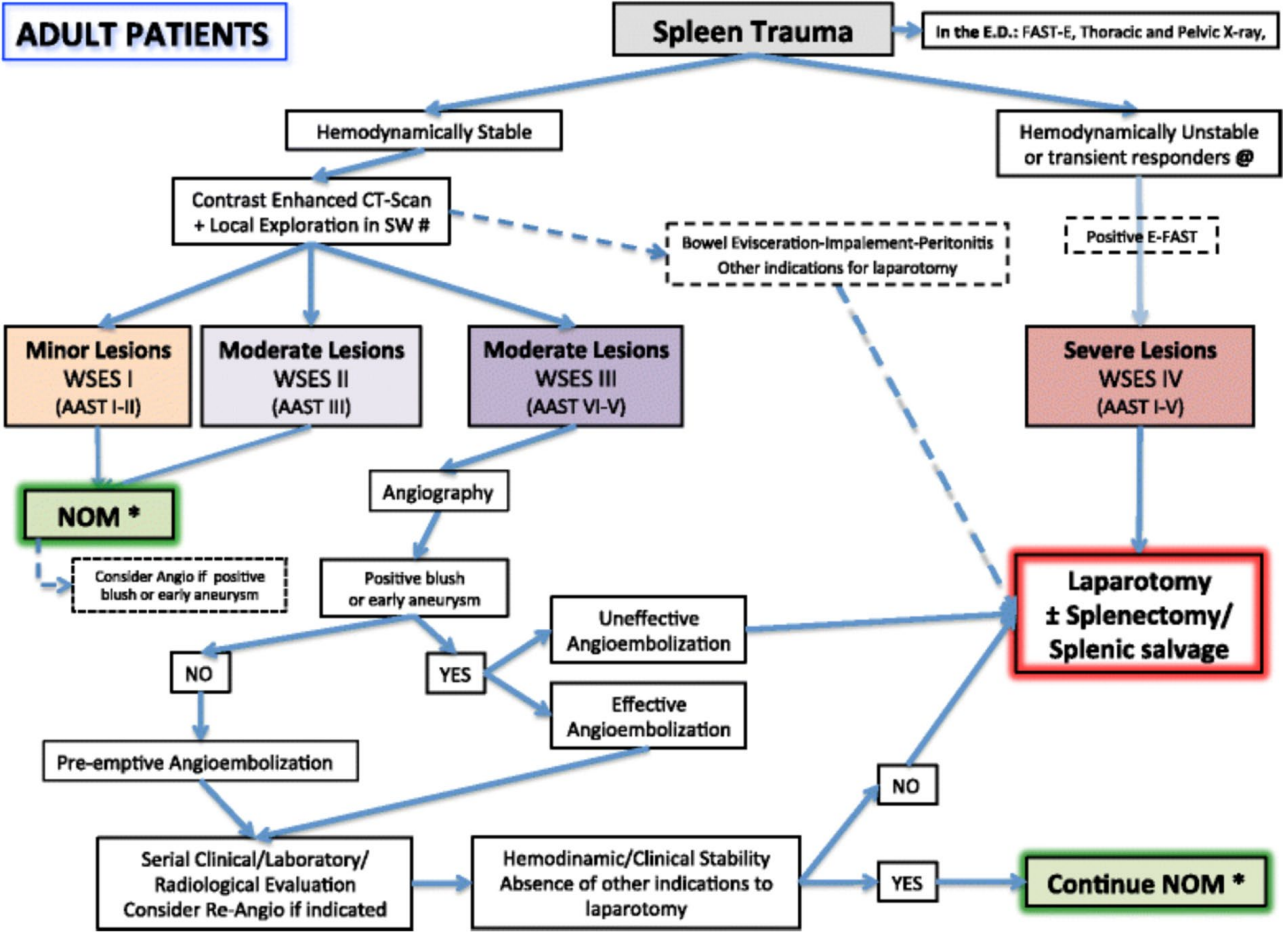
WSES Spleen Trauma Management Algorithm for Adult Patients (*WSES* World Society of Emergency Surgery; *SW* stab wound; *GSW* gunshot wound. *NOM should only be attempted in centers capable of a precise diagnosis of the severity of spleen injuries and capable of intensive management (close clinical observation and hemodynamic monitoring in a high dependency/intensive care environment, including serial clinical examination and laboratory assay, with immediate access to diagnostics, interventional radiology, and surgery and immediately available access to blood and blood products or alternatively in the presence of a rapid centralization system in those patients amenable to be transferred; @ Hemodynamic instability is considered the condition in which the patient has an admission systolic blood pressure < 90 mmHg with evidence of skin vasoconstriction (cool, clammy, decreased capillary refill), altered level of consciousness and/or shortness of breath, or > 90 mmHg but requiring bolus infusions/transfusions and/or vasopressor drugs and/or admission base excess (BE) >  − 5 mmol/l and/or shock index > 1 and/or transfusion requirement of at least 4–6 units of packed red blood cells within the first 24 h; moreover, transient responder patients (those showing an initial response to adequate fluid resuscitation, and then signs of ongoing loss and perfusion deficits) and more in general those responding to therapy but not amenable of sufficient stabilization to be undergone to interventional radiology treatments. # Wound exploration near the inferior costal margin should be avoided if not strictly necessary because of the high risk to damage the intercostal vessels) cited from in Coccolini F et al. [9]

The study was performed following the current publication guidelines of the TR DGU and is registered as the TR-DGU Project ID 2021–015. Since the study was a retrospective analysis based on aggregated routine data, ethics approval was not needed according to the responsible regional medical association (Fig. 1).

### Analysis

**Fig.2:**
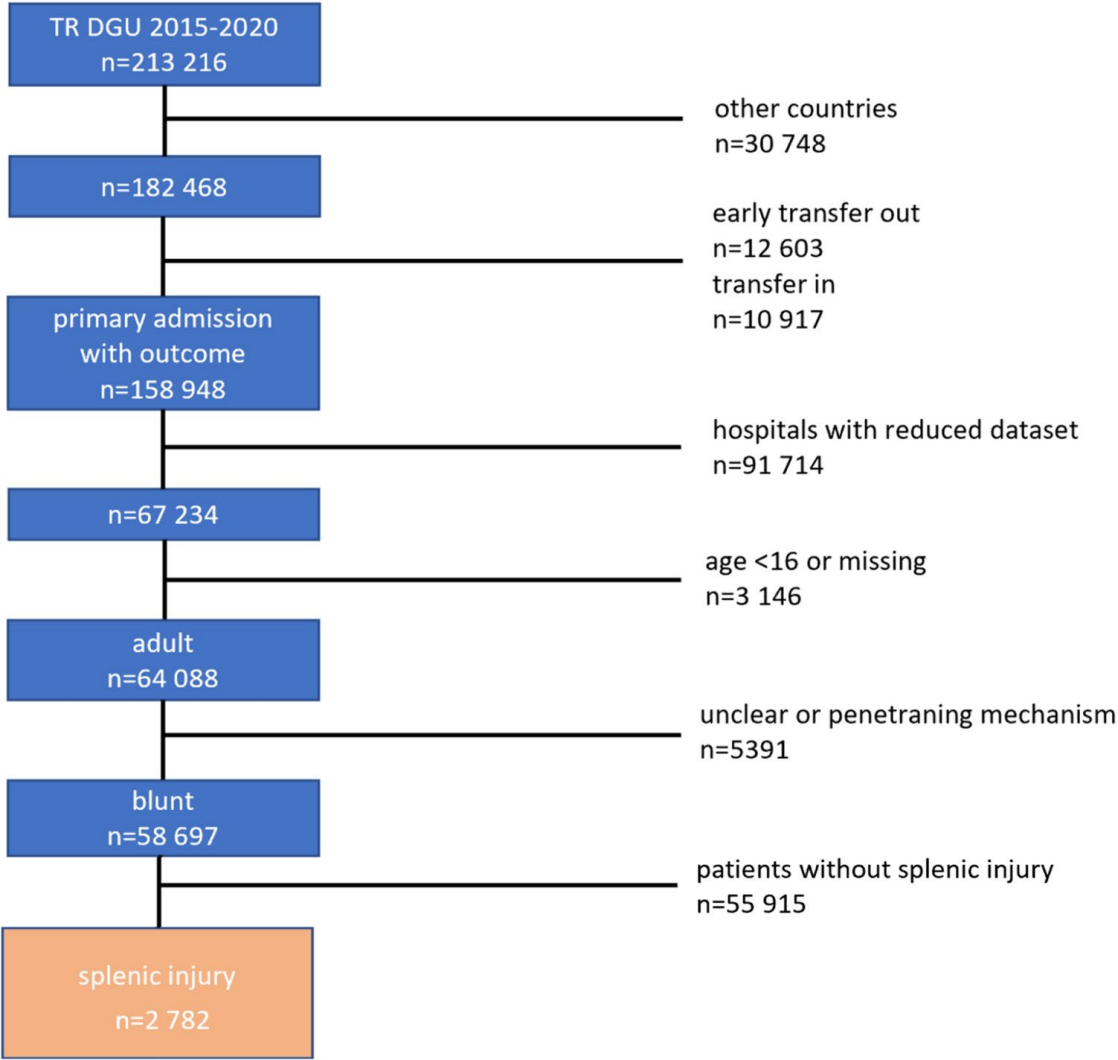
Flowchart of the systematic analysis of the “TraumaRegister DGU® database” with criteria for patient inclusion and exclusion

A retrospective systematic analysis of patients with BSI in the database of the TR DGU was performed. We included trauma patients with documented blunt splenic injuries of AIS Severity 2 and above in the TR DGU standard documentation cohort, which were treated in a resuscitation room and spend more than 24-h in an intensive care unit or died in the resuscitation room. Only patients ≥ 16 years of age, who were primarily admitted to a German trauma center were considered. Patients with penetrating injuries, patients < 16 years of age, patients treated in hospitals with a reduced dataset (limited information about the surgical approach), and patients who were transferred in the initial phase of trauma care were excluded. The period was limited from 2015 to 2020 (Fig. 2).

Demographic data (age, sex) and relevant concomitant injuries (head injury AIS ≥ 3, thoracic injury AIS ≥ 3 and extremity injury AIS ≥ 3) were collected to control for potential confounders that could influence the differences between treatment groups.

For all patients with BSI, we assessed whether NOM or OM was performed and whether NOM was augmented by ANGIO according to their AAST OIS and WSES STC. (Classifications shown in Tables 1 and 2).

To retrospectively classify patients into WSES classes with the data from the National Trauma Registry, hemodynamic instability (INSTBL) needed to be assessed. According to the definitions used by the WSES, patients were classified as INSTBL if the following was documented at emergency room arrival [9, 17]:

- Blood pressure (BP) ≤ 90 mmHg
- Shock Index (SI) > 1
- Base excess (BE) < -5

### Statistical tests and methods

Differences between patients who underwent NOM and those who underwent OM were tested using the chi-square test or Fisher´s exact test. For categorical and continuous data, the Mann‒Whitney U test was used.

To evaluate the influence of splenic injury severity according to the AAST-OIS on overall mortality, the incidence of OM and splenectomy were compared, and the chi-square test was used to determine the trend. For all tests performed, a p value < 0.05 was considered to indicate statistical significance.

All calculations were performed using SPSS Statistics Version 25 (IBM, Armonk, NY, USA), and figures were created using Excel Office 365 (Microsoft Corp., Redmond, WA, USA).

## Results

### Demographic and clinical data

A total of 2782 patients with BSI met the inclusion criteria and were enrolled in the analysis. The mean overall age was 45.7 years, and the population was predominantly male. Neither of these demographic parameters significantly differed between the NOM and OM groups.

Mean injury severity score (ISS) was 29.4 ± 14.9 and significantly higher in the OM group suggesting higher severity and less isolated injuries in those who have been managed surgically, while concomitant injuries AIS ≥ 3 of the thorax and extremities were not significantly different. Severe head trauma was more common in the NOM group.

Organ injury severity according to AAST grade II was present in 1478 patients (53.1%), which was the most common among all AAST organ injury grades. AAST grades III, IV and V were 22.5%, 16.7% and 7.7%, respectively, of the overall study population. Among the NOM patients, 79.4%, 16.3%, 3.4% and 1.0% had AAST grades II, III, IV and V, respectively. In the OM group, 18.8%, 30.6%, 34.1% and 16.5% of the AASTs were Grade II, III, IV and V, respectively.

According to the WSES Spleen Trauma Classification, 36.3% of the overall population were class I, 14.4% were class II, 12.4% were class III and 36.8% were class IV.

A total of 616 patients (22.2%) required transfusions (pRBCs), and among them, 166 patients (6.0%) received a mass transfusion (≥ 10 pRBCs). The transfusion requirements were significantly greater in the OM group.

The estimated risk of death based on the Revised Injury Severity Classification II (RISC II) was 14.8%. (NOM group, 13.4%; s.OM group, 16.6%). The overall hospital mortality rate was 15.0% (NOM group, 13.9%; OM group, 16.4%). The demographic data, clinical parameters and p values are shown in Table 3.

**Table 3:** Demographics and clinical data of patients with blunt splenic injuries

Demographics and Clinical Data				
Parameter	Overall (n = 2782)	NOM (n = 1575)	OM (n = 1207)	p-value*
Age [mean ± SD]	45.7 ± 20.8	46.0 ± 21.0	45.4 ± 20.5	0.70
Male, n [%]	2005 [72.1]	1115 [70.8]	890 [73.7]	0.088
ISS (mean ± SD)	29.4 ± 14.9	26.5 ± 14.1	33.2 ± 15.1	< 0.001
AAST Organ Injury Grade				
AAST II, n [%]	1478 [53.1]	1,251 [79.4]	227 [18.8]	< 0.001
AAST III, n [%]	625 [22.5]	256 [16.3]	369 [30.6]	< 0.001
AAST IV, n [%]	465 [16.7]	53 [3.4]	412 [34.1]	< 0.001
AAST V, n [%]	214 [7.7]	15 [1.0]	199 [16.5]	< 0.001
WSES Spleen Trauma Class				
I, n [%]	1011 [36.3]	885 [56.2]	126 [10.4]	< 0.001
II, n [%]	401 [14.4]	170 [10.8]	231 [19.1]	< 0.001
III, n [%]	345 [12.4]	31 [2.0]	314 [26.0]	< 0.001
IV, n [%]	1025 [36.8]	489 [31.0]	536 [44.4]	< 0.001
Blood transfusion				
1–9 pRBC, n [%]	616 [22.2]	230 [14.6]	386 [32.1]	< 0.001
Mass transfusion ≥ 10 pRBC, n [%]	166 [6.0]	39 [2.5]	127 [10.6]	< 0.001
Systolic blood pressure ≤ 90 mmHg, n [%]	739 [26.6]	353 [22.4]	386 [32.0]	< 0.001
Concomitant injuries (AIS ≥ 3):				
Head, n [%]	781 [28.1]	472 [30.0]	309 [25.6]	0.012
Thorax, n [%]	1926 [69.2]	1081 [68.6]	845 [70.0]	0.46
Extremities, n [%]	965 [34.7]	559 [35.5]	406 [33.6]	0.32
Liver, n [%]	209 [7.5]	73 [4.6]	136 [11.3]	< 0.001
Other abdominal, n [%]	451 [16.2]	165 [10.5]	286 [23.7]	< 0.001
Mechanism of injury				
Motorvehicle accident, n [%]	977 [35.2]	580 [36.9]	397 [32.9]	0.031
Motorbike accident, n [%]	658 [23.7]	339 [21.6]	319 [26.5]	0.003
Bicycle accident, n [%]	182 [6.6]	96 [6.1]	86 [7.1]	0.276
Pedestrian-vehicle accident, n [%]	135 [4.9]	78 [5.0]	57 [4.7]	0.780
High fall, n [%]	403 [14.5]	236 [15.0]	167 [13.9]	0.394
Low fall, n [%]	241 [8.7]	143 [9.1]	98 [8.1]	0.372
Risk of death based on RISC II Score	14.8%	13.4%	16.6%	< 0.001
Hospital mortality rate	417 [15.0]	219 [13.9]	198 [16.4]	0.069
Complications				
Death due to hemorrhage, n [%]	80 [2.9]	38 [2.4]	42 [3.5]	0.119
Sepsis, n [%]	247 [8.9]	110 [7.0]	137 [11.4]	< 0.001
Multi organ failure, n [%]	764 [27.5]	372 [24.6]	392 [32.5]	< 0.001
Thromboembolic event, n [%]	131 [4.7]	63 [4.0]	68 [5.6]	0.058
Coagulopathy, n [%]	600 [21.6]	272 [17.3]	328 [27.2]	< 0.001

*AAST* American Association for the Surgery of Trauma, *AIS* Abbreviated Injury Score, *BP* blood pressure, *ISS* Injury Severity Score, *pRBC* packed red blood cells, *SD* standard deviation, *RISC II* Revised Injury Severity Score II [33], *Coagulopathy* Quick ≤ 60% or PTT ≥ 40 s. or INR ≥ 1.4, detailed definitions of all criteria can be found in Supplement 1

*Chi-Square-Test

### Frequency of ANGIO utilization and outcomes after BSI

Twenty-eight cases of ANGIO utilization for BSI have been documented since 2016, with a trend toward additional interventions since 2018. The ANGIO rate was 1.0% of the overall BSI population. There were 3 deaths in the ANGIO group, with a mortality of 10.7%, compared to 15.0% overall mortality.

Failure of NOM was defined as documentation of a splenectomy after ANGIO and occurred in 2 out of 28 ANGIO patients (7.1%). (Fig. 2).

### Rate of NOM and ANGIO vs. OM and splenectomy according to the AAST-OIS

The incidence of NOM vs. OM was correlated with the severity of splenic injury according to the AAST grade. A total of 15.4% of AAST Grade II injuries were managed operatively, whereas 59.0% of AAST Grade III injuries, 88.6% of AAST Grade IV injuries and 93.0% of AAST Grade V injuries were managed operatively.

**Fig.3:**
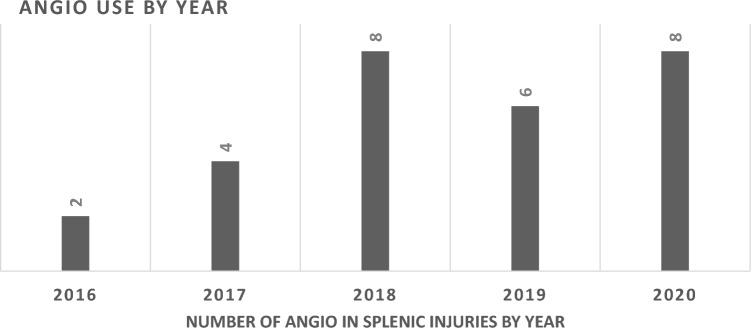
Number of ANGIO = Angiography ± Embolization for patients with blunt splenic injuries within the TraumaRegister DGU® database by year 2016–2020

High-grade splenic injuries are often defined as AAST III–V, 74.2% of which have been managed surgically (Fig. 3).

The rate of splenectomy correlated with injury severity and OM for splenic trauma. For AASTs II, III, IV and V, the rates were 2.6%, 31.6%, 78.7% and 85.5%, respectively. High-grade splenic injuries AAST III–V led to a 58.5% chance of splenectomy.

**Fig.4:**
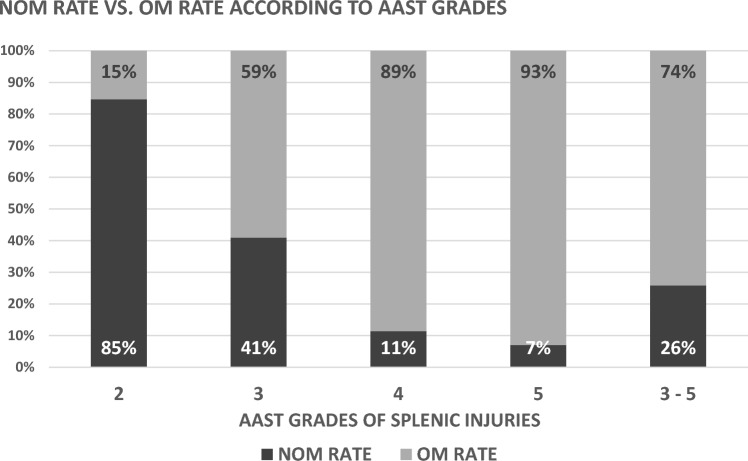
Rate of non-operative management (NOM) vs. operative management (OM) for patients with blunt splenic injuries within the TraumaRegister DGU® database by year 2015–2020

**Fig.5:**
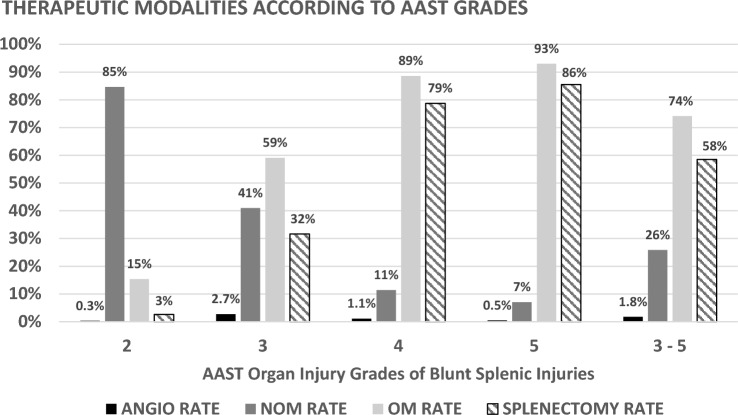
Therapeutic modalities according to the American Association for the Surgery of Trauma (AAST) Organ Injury Grade: Rate of ANGIO = Angiography ± Embolization, rate of NOM = nonoperative management and rate of OM = operative management for patients with blunt splenic injuries within the TraumaRegister DGU® database by year 2015–2020

ANGIO rates were 0.3% for AAST Grade II, 2.7% for AAST Grade III, 1.1% for AAST Grade IV and 0.5% for AAST Grade V. Additionally, 1.8% of high-grade splenic injuries (AAST III–V) involved ANGIO. (Figs. 4 and 5).

### Rate of NOM vs. OM vs. ANGIO according to WSES spleen trauma classification

The incidence of NOM vs. OM was correlated with the severity of splenic trauma according to the WSES class. A total of 12.5% of WSES class I injuries were managed operatively, whereas 57.6% of WSES class grade II injuries, 91.0% of WSES class III injuries and 52.3% of WSES class IV injuries were managed operatively.

The rate of splenectomy correlated with injury severity and OM for splenic trauma. For WSES classes I, II, III and IV, the rates were 2.7%, 29.4%, 81.7% and 35.0%, respectively.

**Fig.6:**
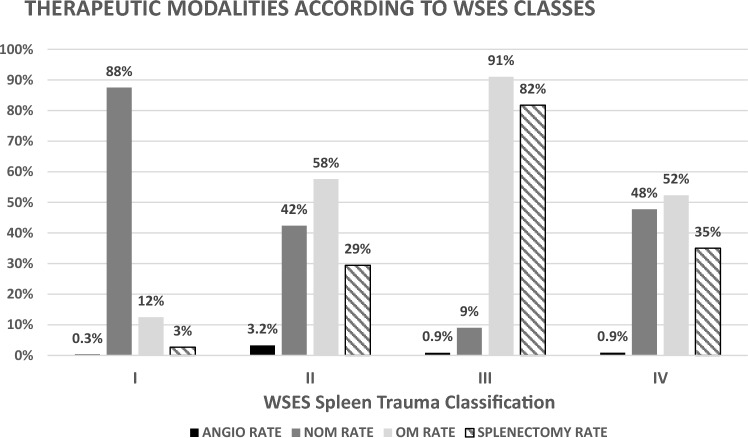
Therapeutic modalities according to WSES Spleen Injury Classes, WSES = World Society of Emergency Surgery: Rate of ANGIO = Angiography ± Embolization, Rate of NOM = Nonoperative Management and Rate of OM = Operative Management for Patients with blunt splenic injurieswithin the TraumaRegister DGU® database by year 2015–2020

The ANGIO rates were 0.3% for WSES class I, 3.2% for WSES class II, 0.9% for WSES class III and 0.9% for WSES class IV. (Fig. 6).

### Utilization and permeation of ANGIO according to the level of care at the treating trauma center

A total of 169 hospitals within the TR DGU were included in the study. Fourteen of them had at least one ANGIO for BSI between 2015 and 2020. Twelve hospitals utilizing ANGIO were level 1 trauma centers, and 2 were level 2 according to the standards of TraumaNetzwerk DGU®, which means that 16.2% of all level 1 trauma centers and 3.5% of all level 2 trauma centers performed ANGIO for BSI.

A total of 12.9% of all level 1 trauma centers performed more than 1 ANGIO for BSI during the study period, and one hospital performed 5 ANGIO during that time. (Table 4).

**Table 4:** ANGIO = Angiography ± Embolization and outcomes after BSI = blunt splenic injuries within hospitals of the TraumaRegister DGU® database by year 2015–2020, DGU = Deutsche Gesellschaft für Unfallchirurgie, failure to NOM = nonoperative management defined as documentation of splenectomy after ANGIO

ANGIO and outcomes after BSI	
Parameter	
Patients with BSI	
With ANGIO, n [%]	28
Without ANGIO, n [%]	2754
ANGIO rate [%]	1.0
In hospital mortality after BSI	
With ANGIO, n [%]	3 [10.7]
Without ANGIO, n [%]	414 [15.0]
Failure to NOM with ANGIO	
n [%]	2 [7.1]

23 ANGIO was performed in patients admitted between 6 am and 6 pm, and 5 ANGIO was performed in patients admitted between 6 pm and 6 am. As 64% of all BSI patients were admitted during the day, this resulted in an ANGIO rate of 1.3% during the day and 0.5% at night. (Table 5).

**Table 5:** Utilization and permeation of ANGIO = Angiography ± Embolization for BSI = blunt splenic injury within hospitals of the TR DGU = TraumaRegister DGU® database by year 2015–2020, DGU = Deutsche Gesellschaft für Unfallchirurgie, *Chi-square test

Utilization and permeation of ANGIO	
Parameter	
Number of hospitals within the analyzed dataset, n	169
Number of hospitals performing ANGIO for BSI according to level of care [%]	
Trauma centers level I, n [%]	12 [16.2]
Trauma centers level II, n [%]	2 [3.5]
Trauma centers level III, n [%]	0 [0]
Level 1 Trauma centers performing > 1 ANGIO 2015–2020	
n [%]	8 [10.9]
Number of ANGIO [rate in % of overall population] according to daytime:	
Between 6am and 6 pm, n [%]	23 [1.3]
Between 6 pm and 6am, n [%]	5 [0.5]

## Discussion

The aim of our study was to analyze the frequency of ANGIO for blunt splenic injuries in Germany since the revision of the 2016 National Trauma Guideline recommendation and outcomes.

Despite the trend toward greater ANGIO utilization since 2018, the overall number of patients was extremely low.

Of the 169 participating trauma centers, ANGIO was performed for BSIs in only 14 centers within the 6-year study period, and more than one was performed in only 8 hospitals. These findings suggest that ANGIO is not a well-established therapy for BSIs at most German level 1 trauma centers. There is no data specifically analyzing the utilization of ANGIO for BSI before 2015 on a nationwide scale, which can be explained by the lack of an ANGIO specific item on the Data entry form of TR DGU.

The overall ANGIO rate was low, at only 1.0%. During the nighttime, it was only 0.5%, possibly reflecting a lack of permeation of guideline recommendations, organizational challenges and a shortage of interventionalists for performing this procedure [34–36]. The availability and total number of radiographic interventions is constantly rising during the last years in Germany, but this is mostly driven by the elective setting [37]. The 24/7 availability of emergency interventional radiography is mandatory for certification as a level 1 trauma center or stroke-unit in Germany, but it is a topic of discussion how those interventions can be offered widely in the emergency setting in the future [38].

Regarding international ANGIO utilization, there is a wide dispersion of institutions, even in health systems with great resources such as Japan [39, 40] or the United States, where published ANGIO rates range from 1 to 19% [6, 13, 41, 42], while good functional outcomes were not exclusively reported by centers with the highest rates of ANGIO [43]. This again shows that patient selection is the key to success in the management of those patients.

As expected, the severity of injury in the OM group was greater for ISS, AAST OIS, hemodynamic instability, transfusion requirements and predicted mortality according to the RISC II score (including 15 items; for details, see Supplement S1).

The incidence of traumatic brain injury (TBI) was greater in the NOM group. Judicious patient selection is mandatory because patients who do not tolerate hypotension resulting from hemorrhage in the case of FNOM can have increased mortality and poor functional outcomes [44]. Nonetheless, if treated in experienced centers, patients with TBI and BSI can be safely and successfully managed nonoperatively [45–47].

The given splenectomy rate of 58.5% in our population for AAST Grade III-IV injuries is among the highest in the literature, compared to the splenectomy rates of approximately 20% in other recent publications [6, 13, 15, 42]. We validated our findings by cross checking the ANGIO and splenectomy rate with five major German trauma centers and found no evidence of a systemic error within our study data.

Mortality was lower in the ANGIO group. However, whether this reflects a lower grade of initial hemodynamic instability, a real treatment effect of ANGIO or mere chance cannot be derived from these data. Failure of NOM occurred in only 2 patients, but this finding is also limited by the small number of interventions performed during the study period.

As the WSES Spleen Trauma Classification includes clinical features of instability, we also examined the distribution of therapeutic modalities according to this classification to compare it with the WSES guideline recommendations on splenic trauma [9].

The majority of WSES class I patients have been managed with NOM, as suggested by the guidelines.

For class II patients, which should also be managed nonoperatively according to the WSES, we already observed an OM rate of 58%. As the OM group had significantly more concomitant abdominal injuries, laparotomies were performed for other indications, but a splenectomy rate of 29% for WSES class II suggested deviation from the guideline recommendations. WSES class III patients were predominantly managed operatively and underwent splenectomy; the ANGIO rate was only 0.9% in this group, while the WSES recommendations emphasize the role of ANGIO, especially for those patients.

Interestingly, nearly half of the WSES class IV patients were successfully managed nonoperatively. All these patients had signs of hemodynamic instability as defined by the WSES; therefore, operative management was recommended [9, 17]. The lower rate of OM and splenectomy in WSES class IV compared to class III might be explained by the definition with WSES class III being based on anatomical severity of injury, WSES class IV includes also lower anatomical severity of injury with signs of instability as demonstrated in Table 2.

Organ salvage is a therapeutic goal worth pursuing, as splenectomy has a considerable impact on immunity and other organ systems. Overwhelming post splenectomy infection (OPSI) is a feared complication that can occur after splenectomy [48]. It was also demonstrated that splenectomy is an independent risk factor for infectious complications such as intra-abdominal abscess, wound infection, pneumonia, and septicaemia [49]. Additionally, splenectomy causes a permanent hypercoagulable state, venous thromboembolism and an increased incidence of subsequent malignant disease [50–52].

On the other hand, ANGIO is associated with increased morbidity after NOM for BSI; reported complications include splenic abscess, infarction, pseudocyst or vascular injury, pancreatitis, intestinal perforation, abdominal compartment syndrome, acute respiratory distress syndrome and multiorgan failure [27, 53–55]. It was a concern of surgeons that ANGIO was basically a form of functional splenectomy performed at another location. This notion can be contradicted by the meta-analysis of Schimmer et al., which showed preserved splenic function after both selective and proximal ANGIO [56]. This difference might be explained by preserved arterial blood flow to the spleen via the short gastric vessels.

This cross-sectional and longitudinal study evaluated a high number of severely injured patients and trauma centers. Overall mortality was high (15.0%), which is likely and substantially influenced by concomitant injuries. In particular, liver and other abdominal injuries were significantly more common in the OM group than in the control group.

This study is limited by its retrospective design and reliance on the quality of the documentation in the database. The German national trauma guideline recommends ANGIO in patients who are “stabilizable”. Our data do not allow us to retrospectively distinguish between "responders" and "nonresponders” to resuscitation, which impacts therapy selection between NOM ± ANGIO and OM [57]. Furthermore, it is not reliably possible to differentiate between primary ANGIO or secondary ANGIO as an adjunct to NOM.

## Conclusion

BSI is associated with a significant mortality rate. In Germany, ANGIO was rarely used to augment NOM, in contrast to national and international guideline recommendations. Surgical treatment remains predominant for splenic injuries with an AAST ≥ 3, even if the patient’s hemodynamic status might allow for a course of NOM.

The given splenectomy rate in this study is a call to salvage more organs. Trauma centers might implement and train local treatment algorithms, including 24/7 ANGIO capabilities and criteria which patients should be augmented with ANGIO based on current guidelines like the German national trauma guideline and international recommendations like the WSES Spleen Trauma Management Algorithm. Those local protocols must consider the institutions specifics and might be beneficial to improve organ salvage rates in German trauma centers in the future.

## Supplementary Information

Below is the link to the electronic supplementary material.

Supplementary file1 (PDF 606 KB)

## Data Availability

No datasets were generated or analysed during the current study.

## Abbreviations

AAST OIS: American Association for the Surgery of Trauma Organ Injury Scale
AIS: Abbreviated Injury Score
ANGIO: Angiography ± Embolization
BP: Blood pressure
BSI: Blunt splenic injury
DGU: Deutsche Gesellschaft für Unfallchirurgie
ISS: Injury Severity Score
pRBC: Packed red blood cells
NOM: Nonoperative management
RISC II: Revised Injury Severity Score II
SD: Standard deviation
TR DGU: TraumaRegister DGU®
WSES: World Society of Emergency Surgery
