# Supplementary material for: Angioembolization in patients with blunt splenic trauma in Germany –guidelines vs. Reality a retrospective registry-based cohort study of the TraumaRegister DGU®
Source: Eur J Trauma Emerg Surg. 2024 Sep 16;50(5):2451–62. doi: 10.1007/s00068-024-02640-6 (PMC11599407; doi:10.1007/s00068-024-02640-6)
Supplement: Supplementary file 1 — Supplementary file1 (PDF 606 KB) [file 68_2024_2640_MOESM1_ESM.pdf]

TraumaRegister DGU®
DEUTSCHE GESELLSCHAFT FÜR UNFALLCHIRURGIE

Leitfaden zum Ausfüllen

des Erhebungsbogens des

TraumaRegister DGU®

Version 1.5 für V2020

30. Juni 2020

Inhalt

Versionshistorie ... 7
Allgemeine Anmerkungen ... 8
Einschlusskriterium ... 8
Erhebungsbögen ... 8
Standardbogen ... 8
QM-Bogen für das TraumaNetzwerk DGU® ... 8
Parameter-Darstellung im Leitfaden ... 8
Bogenrevisionen ... 9
Support/Hotline ... 10
Bogen S – Stammdaten ... 11
Patienten-Code ... 11
Interne Bemerkung ... 11
Box Geschlecht ... 11
Geschlecht ... 11
Wenn weiblich, bestand eine Schwangerschaft ... 11
Box Unfall-Anamnese ... 11
Unfallzeitpunkt ... 11
Box Zuverlegt ... 11
Zuverlegt ... 11
Box Gesundheitszustand vor Unfall ... 12
ASA vor Unfall ... 12
Antikoagulation ... 13
Box Unfallart ... 13
Verkehr ... 13
Stürze ... 14
Sonstige Unfallursache ... 14
BG-/AUVA-Fall ... 15
Box Bestätigung Patienteneinwilligung ... 15
Bestätigung Patienteneinwilligung (nicht abgebildet auf PDF-Erhebungsbogen) ... 15
Box Module des TR-DGU ... 15
Module des TR-DGU (nicht abgebildet auf PDF-Erhebungsbogen) ... 15
Bogen A – Präklinik ... 15

© 2020 – AUC - Akademie der Unfallchirurgie GmbH / Sektion NIS

Box Rettungszeiten ... 15
Erste Alarmierung ... 15
Ankunft erstes Rettungsmittel Unfallstelle ... 15
Transportbeginn mit Patienten ... 15
Notärztliche Behandlung am Unfallort ... 15
Transport ... 15
Nachforderung RTH ... 16
MANV ... 16
Box Vitalzeichen ... 16
Kapnometrie durchgeführt ... 16
Glasgow Coma Scale ... 16
Pupillenweite ... 17
Lichtreaktion ... 17
Box Verletzungen ... 17
Box Therapie ... 17
Endotracheale Intubation ... 17
Chirurgischer Atemweg ... 17
Alternative Atemwegssicherung ... 17
HWS-Immobilisierung ... 17
Nadeldekompression ... 18
Thoraxdrainage ... 18
Beckengurt ... 18
Tourniquet ... 18
IO-Zugang ... 18
Herzdruckmassage ... 18
Präklinische Thorakotomie ... 18
Tranexamsäure ... 18
Katecholamine ... 18
Analgosedierung ... 18
Bogen B – Schockraum- und OP-Phase ... 19
Box Aufnahme ... 19
Box COVID-19 ... 19
Box Vitalzeichen ... 19
Herzfrequenz ... 19
Bereits bei Ankunft beatmet? ... 19
FiO_2 ... 19

PaO_2 19
Glasgow Coma Scale 20
Pupillenweite 20
Lichtreaktion 20
Box Labor 20
Hämoglobin 20
Thrombozytenzahl 20
TPZ (Quick) 20
PTT 21
INR 21
Base Excess (-/+) 21
Ca (ionisiert) 21
Ethanol (Plasma) 21
Fibrinogen 21
Box Diagnostik 21
FAST / eFAST 21
Freie Flüssigkeit bei FAST? 21
Selektives CT 22
MRT 22
ROTEM® 22
EXTEM-CT 22
EXTEM-MCF 22
FIBTEM-A10 22
Nutzung Teleradiologie? 23
Box Volumengabe 23
Box Therapie Schockraum 23
Endotracheale Intubation 23
Chirurgischer Atemweg 23
Thoraxdrainage 23
Herzdruckmassage 23
Beckengurt 23
Box Operative Notfalleingriffe bis Aufnahme ICU/IMC 24
Operative Hirndruckentlastung 24
Laminektomie 24
Thorakotomie 24
Laparotomie 24

Revaskularisation 24
Embolisation 24
REBOA 24
Stabilisierung Becken 24
Stabilisierung Extremitäten 24
Escharotomie 24
Dermatofasziotomie 24
Box Hämostase-Therapie 25
EK-Gabe: Zeitpunkt erste EK-Gabe Uhrzeit 25
Frischplasma (FFP) 25
Thrombozytenkonzentrate 25
Box Medikamentöse Gerinnungstherapie 25
Tranexamsäure 25
Fibrinogen 25
PPSB 25
Kalzium 26
Faktor XIII 26
DOAK-Antidot 26
Box Weiterversorgung 26
Weiterverlegung Uhrzeit 26
Bogen C – Intensivstation 26
Box Labor / Vitalwerte 26
Hämoglobin 26
Thrombozytenzahl 26
Base Excess (-/+) 26
Ca (ionisiert) 26
Fibrinogen 27
Box ROTEM® 27
EXTEM-CT 27
EXTEM-MCF 27
FIBTEM-A10 27
Box Hämostase-Therapie 27
Frischplasma (FFP) 27
Thrombozytenkonzentrat 27
Box Medikamentöse Gerinnungstherapie 27
Tranexamsäure 27

Fibrinogen 28
Faktor XIII 28
Box Aufenthaltsdauer / Beatmungsdauer 28
Intensivtherapie (ohne IMC) 28
Mechanische Beatmung 28
Box Therapie Intensivstation 28
ECMO (Extrakorporaler Lungensupport, Extracorporeal Membrane Oxygenation) 28
Box Organversagen 28
Organversagen 28
MOV 29
Sepsis 29
Wenn Sepsis ja: Fokus? 29
Bogen D – Diagnosen 29
Box Diagnose 29
AIS-Code 29
Beschreibung der Verletzung / Diagnose 30
Diagnosestellungen nach Intensiv-Aufnahme? 30
Box Operation 30
OP-Maßnahme 30
Beschreibung der Operation 30
Box Fraktur 31
Grad der Weichteilverletzung (I-V) 31
Box Initiales Therapiekonzept 31
Bogen D – Abschluss 31
Box Entlassungs-/Todesdatum 31
Box Entlassung/Verlegung/Tod 31
Bei der Angabe „Tod“ 31
Box Zustand bei Entlassung/Verlegung 32
Box Todesursache 32
Todesursache 32
Anhang 1 33

Versionshistorie

Datum	Änderungen
22.06.2017	Redaktionelle Änderungen in Kapitel „Box Outcome“ (S. 18)
04.01.2018	Korrektur Rechtschreibung
19.06.2018 (Version 1.4)	Inhaltliche Änderung Bogen B „Box Atmung“:<br>• Bezeichnung Parameter: Anstelle von „Bereits bei Ankunft intubiert?“ jetzt „Bereits bei Ankunft beatmet?“<br>• Hilfetext: Anstelle von „War der Patient bei Einlieferung endotracheal intubiert“ jetzt „War der Patient bei Einlieferung beatmet“
20.02.2019 (Version 1.4)	Inhaltliche Änderung Bogen S „Antikoagulation“:<br>• Es ist jetzt eine Mehrfachauswahl möglich<br>Inhaltliche Änderung Bogen D „OPS-Code“<br>• Die Möglichkeit zur Angabe des OPS-Codes wurde herausgenommen und gegen eine Liste möglicher Operationen ausgetauscht (OP-Gruppen)<br>Ergänzung um Anhang 1 (Auflistung OP-Gruppen)
Juni 2020 (Version 1.5)	Einarbeitung Änderungen für die BogenRevision 2020<br>(neue und geänderte Parameter sind entsprechend markiert)<br>Gelöschte Parameter: Präklinik: NACA-Index; ICU: Ethanol (Plasma), Kalzium-Gabe

Allgemeine Anmerkungen

Dieser Leitfaden erläutert das Ausfüllen des Erhebungsbogens des TraumaRegister DGU® in der Version V2020, der im Juli 2020 veröffentlicht wurde. Die Live-Schaltung der Online-Masken erfolgte zum 1. Juli 2020.

Einschlusskriterium

Einzuschließen sind alle Patienten, die mit Lebenszeichen über den Schockraum aufgenommen und anschließend eine Intensivtherapie benötigen bzw. vor Aufnahme auf die Intensivstation versterben. Zudem sind alle Patienten einzugeben, die im Rahmen des TraumaNetzwerk DGU® in ein Krankenhaus einer höheren Versorgungsstufe verlegt werden. Für jeden Patienten, der eingegeben wird, muss nach aktueller EU-Gesetzesauslegung (DSGVO) eine Einwilligungserklärung vorliegen. Hierfür liegt ein standardisierter Aufklärungsbogen im TraumaRegister DGU unter „Informationen" -> „Patienteninformation" zum Download bereit.

Erhebungsbögen

Standardbogen

Der Erhebungsbogen des TraumaRegister DGU® liegt in zwei Versionen vor. Der Standardbogen wurde mit Gründung des Registers entwickelt und verlangt die Eingabe von ca. 100 Parametern. Daneben liegt für das TraumaNetzwerk DGU® eine gekürzte Version mit ca. 40 Parametern vor, der sogenannte QM-Bogen (siehe unten).

Der Erhebungsbogen besteht aus fünf Einzelbögen, die zu vier festgelegten Zeitintervallen ausgefüllt werden:

- Bogen S: Stammdaten
- Bogen A: Unfall bis Einlieferung ins Krankenhaus
- Bogen B: Aufnahme Schockraum und ggf. anschließende OP-Phase bis Aufnahme ICU bzw. frühe Weiterverlegung
- Bogen C: Intensivstation
- Bogen D: Zustand bei Entlassung und Diagnosen

QM-Bogen für das TraumaNetzwerk DGU®

Die für das TraumaNetzwerk DGU® verfügbare Version des Erhebungsbogens, der sogenannten QM-Bogen, ist eine gekürzte Version des Standardbogens (ca. 40 Parameter). Der QM-Bogen folgt in seinem Aufbau dem Standardbogen mit der gleichen Seitenanzahl. Dort sind alle Parameter, die nicht beim QM-Bogen erfasst werden sollen, ausgegraut. Diese Darstellung findet sich auch in den Masken der Online-Eingabe wieder.

Parameter-Darstellung im Leitfaden

Auf den folgenden Seiten wird das Ausfüllen des Standardbogens erläutert. Dabei werden alle Parameter genannt, die auch in der Online-Eingabe mit einem Hilfetext versehen sind bzw. die mit der Datensatz-Revision V2015 neu hinzugekommen sind.

Der Erhebungsbogen besteht aus dem fünf Einzelbögen (S – A – B – C – D, siehe oben), die im Weiteren „Bogen" genannt werden. Innerhalb dieser Bögen sind die Parameter thematisch in unterschiedlichen Boxen gelistet, siehe Abb. 1.

Die Auflistung der Parameter folgt dem Aufbau des Standardbogens, beginnend mit „Bogen S – Stammdaten“. Es werden erst die Parameter der linken Spalte und dann die der rechten Spalte erläutert (siehe Papier-Version des Erhebungsbogens bzw. Online-Masken).

Der Aufbau der Papierversion des Erhebungsbogens und der Online-Masken wurde so weit wie möglich angeglichen.

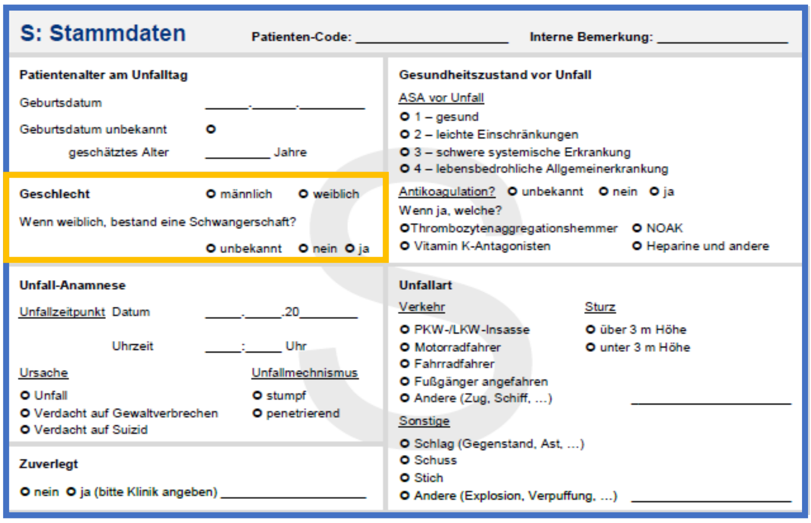
S: Stammdaten Patienten-Code: ________ Interne Bemerkung: ________

Patientenalter am Unfalltag
Geburtsdatum ____.____.______
Geburtsdatum unbekannt O
geschätztes Alter ________ Jahre

Geschlecht O männlich O weiblich
Wenn weiblich, bestand eine Schwangerschaft?
O unbekannt O nein O ja

Unfall-Anamnese
Unfallzeitpunkt Datum ____.____.20____
Uhrzeit ____:____ Uhr
Ursache
O Unfall
O Verdacht auf Gewaltverbrechen
O Verdacht auf Suizid
Unfallmechnismus
O stumpf
O penetrierend

Zuverlegt
O nein O ja (bitte Klinik angeben) ________

Gesundheitszustand vor Unfall
ASA vor Unfall
O 1 – gesund
O 2 – leichte Einschränkungen
O 3 – schwere systemische Erkrankung
O 4 – lebensbedrohliche Allgemeinerkrankung
Antikoagulation? O unbekannt O nein O ja
Wenn ja, welche?
O Thrombozytenaggregationshemmer O NOAK
O Vitamin K-Antagonisten O Heparine und andere

Unfallart
Verkehr
O PKW-/LKW-Insasse
O Motorradfahrer
O Fahrradfahrer
O Fußgänger angefahren
O Andere (Zug, Schiff, …) ________
Sturz
O über 3 m Höhe
O unter 3 m Höhe
Sonstige
O Schlag (Gegenstand, Ast, …)
O Schuss
O Stich
O Andere (Explosion, Verpuffung, …) ________

Abbildung 1 Exemplarische Darstellung eines Einzelbogens: "Bogen" blau umrahmt, "Box" orange umrahmt.

Weitere Einzelheiten zum TraumaRegister DGU® können der Homepage entnommen werden: www.traumaregister-dgu.de.

Dort sind auch die Erhebungsbögen verfügbar: http://www.traumaregister-dgu.de/index.php?id=1445&L=964

Der Online-Zugang zur Eingabe ins TraumaRegister DGU® erfolgt über das TraumaPortal DGU: https://intern.dgu-traumanetz.de/intern/de/login/index.jsp

Bogenrevisionen

Der Datensatz des TraumaRegister DGU® wird in regelmäßigen Abständen einer Revision unterzogen, der sogenannten BogenRevision.

Die BogenRevisionen werden vom „Arbeitskreis TraumaRegister“ (wissenschaftliches Board) der Sektion NIS der DGU gemeinsam mit der „AUC – Akademie der Unfallchirurgie GmbH“ (Betreiber) erarbeitet.

Diese Revisionen werden momentan alle fünf Jahre parallel und in Abstimmung mit dem „DIVI-Notaufnahmeprotokoll“ durchgeführt (https://www.divi.de/forschung/sektionsgruppen/notfallmedizin/notaufnahmeprotokoll). Die Parameter der Bögen S (Stammdaten), A (Präklinik) und B (Schockraum) des TraumaRegister DGU® sind im „DIVI-Notaufnahmeprotokoll“ enthalten.

Die untenstehende Tabelle gibt die Bezeichnung und die Gültigkeit der letzten Bogenrevisionen an. Die Bogenrevisionen werden mit einem V (für Version) und einer Jahreszahl benannt. Die Versionsnummer ist auf den Papier-/PDF-Erhebungsbögen jeweils rechts oben vermerkt.

BogenRevision	Gültigkeit
V2009	08/2009 bis 01/2016
V2015	01/2016 bis 08/2019
V2015.1	08/2019 bis 06/2020
V2020	07/2020 bis (2025)

Support/Hotline

Weitere Fragen beantwortet die Hotline des TraumaRegister DGU®:

Email: support-tr@auc-online.de

Tel: +49 221 888 239 – 10

Hotline-Zeiten: Mo – Fr 9 bis 12 Uhr, 13 bis 15 h

Bogen S – Stammdaten

Patienten-Code

- Der Patienten-Code setzt sich aus dem Unfalljahr und einem frei vergebbaren Code zusammen.
- Das Unfalljahr wird automatisch ergänzt (wird aus der Angabe des Unfallzeitpunkts generiert).
- Es können nur Zahlen verwendet werden, wobei eine Durchnummerierung mit führenden Nullen entsprechend der erwarteten Patientenzahl empfohlen wird (01, 02, 03, ... bzw. 001, 002, 003, ...).
- Die Durchnummerierung kann jedes Jahr neu beginnen.

Interne Bemerkung

- Sie können für jeden Patienten eine zusätzliche, eindeutige, beliebige Kennung vergeben (nur Zahlen, z.B. KIS-Nr.).

Box Geschlecht

Geschlecht

- Es ist das biologische Geschlecht anzugeben.
- **Neue Kategorie ab V2020:**
  Neue Kategorie „divers“ in Anpassung an die Änderung im Personenstandsrecht in Deutschland.

Wenn weiblich, bestand eine Schwangerschaft

- Lag bei einem weiblichen Patienten eine Schwangerschaft vor?

Box Unfall-Anamnese

Unfallzeitpunkt

- Wenn der Unfallzeitpunkt nicht bekannt ist, dann bestmöglich schätzen.
- Zwischen Unfallzeitpunkt und Aufnahme Schockraum dürfen nicht mehr als 24 h liegen (Ausschlusskriterium bei Primärpatienten).

Box Zuverlegt

Zuverlegt

- Bei zuverlegten Patienten ist das zu verlegende Krankenhaus zu nennen.

Box Gesundheitszustand vor Unfall

ASA vor Unfall

- Angabe des prätraumatischen Allgemeinzustands, wobei ASA 5 und 6 unberücksichtigt bleiben.

ASA-Klassifi-kation	Definition	Beispiele für Erwachsene
**ASA I**	Normaler gesunder Patient	Gesund, Nichtraucher, wenig oder kein Alkohol
**ASA II**	Patient mit leichter systemischer Erkrankung	Leicht erkrankt ohne relevante funktionelle Einschränkungen. Beispiele beinhalten: Raucher, leichte Alkoholiker, Schwangere, Adipositas (30<BMI<40), gut eingestellte Diabetes/arterielle Hypertonie, leichte Lungenerkrankung
**ASA III**	Patient mit schwerer systemischer Erkrankung	Relevante funktionelle Einschränkungen; eine oder mehrere leichte Erkrankungen. Beispiele beinhalten: schnell eingestellter Diabetes/Hypertonie, COPD, Adipositas (BMI≥40), Hepatitis, Alkoholabhängigkeit und -missbrauch, implantierter Schrittmacher, leichte Reduktion des EF, Niereninsuffizienz mit regelmäßiger Dialyse, post-konzeptionelles Alter < 60 Wochen, anamnestische (>3 Monate) MI, Apoplex, TIA, KHK/Stent
**ASA IV**	Patient mit schwerer systemischer Erkrankung mit permanenter Lebensgefahr	Beispiele beinhalten: kürzlicher (< 3 Monate) Herzinfarkt, Apoplex, TIA oder KHK/Stent, fortgesetzte kardiale Ischämie oder schwere Klappenfunktionsstörung, relevante Einschränkung der EF, Sepsis, disseminierte intravasale Koagulopathie, akute respiratorische oder dialysepflichtige Niereninsuffizienz
**ASA V**	Im Sterben liegender Patient, der ohne OP vermutlich nicht überlebt	Beispiele beinhalten: rupturiertes abdominales/thorakales Aneurysma, massives Trauma, intrakranielle Massenblutung mit Mittellinienverlagerung, Darmischämie bei zugrundeliegender kardialer Pathologie oder Multiorganversagen
**ASA VI**	Hirntoter Patient, dessen Organe zur Organspende freigegeben sind	-

Quelle: Nach ASA Houses of Delegates, ASA physical classification system: https://www.asahq.org/standards-and-guidelines/asa-physical-status-classification-system (aufgerufen 27.11.2019)

Antikoagulation

- Wurde der Patient vor Unfall mit Antikoagulanzien behandelt?
- Es ist eine Mehrfachauswahl bei den Medikamenten möglich (seit 05/19)
- **Neue Kategorien ab V2020:**
    - Die Thrombozytenaggregationshemmer wurden unterteilt in „ASS" (Acetylsalicylsäure) und „andere Thrombozytenaggregationshemmer".
    - Die Auswahlmöglichkeiten „Sonstige" und „unbekannt" wurden ergänzt.
- **Umbenannte Kategorien ab V2020:**
    - NOAK in DOAK (Direkte orale Antikoagulantien)
    - „Heparine und andere" in „Heparin(oide)"

Box Unfallart

Neu ab V2020: Die Auswahlmöglichkeiten bei den Kategorien wurden umfassender überarbeitet.

Verkehr

PKW-Insasse

- **Neue Kategorie ab V2020**!
    - jetzt PKW-Insasse als eigenständige Kategorie.
      (war vorher als PKW-/LKW-Insasse in einer Variablen zusammengefasst).
    - Als PKW in diesem Sinne wird ein Fahrzeug bis 7,5t bezeichnet.

LKW-Insasse

- **Neue Kategorie ab V2020**!
    - jetzt LKW-Insasse als eigenständige Kategorie (war vorher als PKW-/LKW-Insasse in einer Variablen zusammengefasst).
    - Als LKW in diesem Sinne wird ein Fahrzeug über 7,5t bezeichnet.

Bus-Insasse

- **Neue Kategorie ab V2020**!
- Gemeint sind verunglückte Personen in Bussen (ÖPNV, Reisebus, etc.).

Motorradfahrer/-sozius

- Gemeint ist der Motorradfahrer selbst oder ein Beifahrer (Person vor/hinter Fahrer oder im seitlich montierten Sozius sitzend).

Fahrrad

- Gemeint sind alle Fahrräder ohne Hilfsmotor.

Unterstütztes Fahrrad

- **Neue Kategorie ab V2020!**
- Gemeint sind alle Arten von elektrisch unterstützen Fahrrädern (E-Bike/(S-)Pedelec), unabhängig von der Bezeichnung und der Stellung in den Straßenverkehrsordnungen dieser Fahrräder in den einzelnen Ländern.

E-Scooter

- **Neue Kategorie ab V2020**!
- Gemeint sind alle Arten elektrischer Tretroller (E-Scooter).

Fußgänger angefahren

- Gemeint sind Fußgänger, die durch einen anderen Verkehrsteilnehmer angefahren wurden.

Sonstiger Verkehrsunfall

- Hier können andere als die oben genannten Verkehrsunfälle über ein Freitextfeld eingetragen werden.

Stürze

Sturz mit Fallhöhe >= 3m

- Gemeint sind alle Stürze aus über drei Meter Höhe (Fenstersturz, Baum, Brücke, etc.).
- Treppenstürze fallen unter die Kategorie „Sturz aus Fallhöhe < 3m“!

Sturz mit Fallhöhe < 3m

- Meint Treppenstürze oder Sturz aus dem Bett.

Ebenerdiger Sturz

- **Neue Kategorie ab V2020**!
- Gemeint ist der Sturz aus dem Stand, z.B. über die Teppich- oder Bordsteinkante.

Sonstige Unfallursache

Schlagverletzung

- Schlag meint jede stumpfe Einwirkung, egal ob durch einen Menschen, Gegenstand oder sonstige Einwirkung von außen (z.B. Gegenstand, Ast, etc.).

Schussverletzung

- Schuss meint jede Verletzung beigebracht durch eine Schusswaffe, egal ob Langwaffe, Kurzwaffe, Schreckschusspistole, Luftgewehr, etc.

Stichverletzung

- Stich meint jede Verletzung beigebracht durch ein Stichwerkzeug, egal ob Messer, Schwert, jegliche andere Klinge, Schraubenzieher, Stock, etc., welches zu einer Perforation führt (also zum Verlust der Schutzfunktion der Haut).

Explosion / Verpuffung

- **Neue Kategorie ab V2020**!
- Jetzt als eigenständige Kategorie (konnte vorher unter „andere Unfallarten“ angegeben werden).
- Gemeint sind thermodynamische Kombinationsverletzungen.

Verschüttung

- **Neue Kategorie ab V2020!**
- Hierunter werden alle Verschüttungstraumata (Sand, Steine, Mauern, Schnee, etc.) subsummiert.

Andere Unfallart

- Hier können andere als die oben genannten Unfallarten über ein Freitextfeld eingetragen werden.
- „Andere“ Verkehrsunfälle bitte bei „Sonstigen Verkehrsunfall“ eintragen!

BG-/AUVA-Fall

- **Neue Variable ab V2020!**
  Nur von deutschen (BG) und österreichischen Kliniken (AUVA) anzugeben.
- Hier sind Patienten gemeint, die arbeitsbedingte Unfälle im Rahmen ihrer gesetzlichen Unfallversicherung erleiden.

Box Bestätigung Patienteneinwilligung

Bestätigung Patienteneinwilligung (nicht abgebildet auf PDF-Erhebungsbogen)

- Für die Eingabe der Daten ist eine Patienteneinwilligung notwendig. Beim Anlegen eines Falles wird das Vorhandensein dieser Einwilligung abgefragt.
- Hier können die beim Anlegen gemachten Angaben geändert werden, z.B. wenn der Patient später verstorben ist.

Box Module des TR-DGU

Module des TR-DGU (nicht abgebildet auf PDF-Erhebungsbogen)

- Für das TR-DGU bestehen inzwischen mehrere zusätzliche Module, die für bestimmte Verletzungsmuster eine detaillierte Dokumentation des Behandlungsverlaufs ermöglichen. Hier sind die Module aufgelistet, an denen das Krankenhaus teilnimmt. Die Module laufen auf der AUC-Registerplattform, für die eigene Zugangsdaten benötigt werden.
- Bitte geben Sie an, für welche(s) Modul(e) weitere Daten eingegeben werden sollen. Mit Abspeicherung dieser Seite werden der Patienten-Code in das Modul in der AUC-Registerplattform übertragen.

Bogen A – Präklinik

Box Rettungszeiten

Erste Alarmierung

- Gemeint ist die erste Alarmierung bei der Leitstelle.

Ankunft erstes Rettungsmittel Unfallstelle

- Ankunftszeit des ersteintreffenden Rettungsmittels an der Unfallstelle.

Transportbeginn mit Patienten

- Beginn (Uhrzeit) des Transports des Patienten von der Unfallstelle ins Krankenhaus.

Notärztliche Behandlung am Unfallort

- **Neue Variable ab V2020!**
- Hier ist anzugeben, ob eine notärztliche Behandlung am Unfallort stattgefunden hat.

Transport

- Bei kombiniertem Transport soll nur das aufwändigere Transportmittel angegeben werden.

Nachforderung RTH

- **Neue Variable ab V2020!**
- Zur Unterscheidung, ob primär der RTH vor Ort war oder nachgefordert wurde durch einen NA oder Rettungsteam vor Ort.

MANV

- **Neue Variable ab V2020!**
- Der „Massenanfall von Verletzten" (MANV) bezeichnet eine Situation, bei der eine große Zahl von Betroffenen versorgt werden muss.
- Die Definition ist uneinheitlich: Daher gilt die örtliche Regelung.
  In Deutschland wird z.B. in den Landesgesetzen auch der Begriff „Großschadenslage" synonym verwendet.

Box Vitalzeichen

Es sollen die von der Therapie unbeeinflussten Werte beim Eintreffen des ersten Rettungsmittels erfasst werden.

Neu ab V2020:
Die Reihenfolge der Maßnahmen wurde an die Reihenfolge im ATLS-Schema angepasst!

Kapnometrie durchgeführt

- Wurde beim intubierten Patienten eine Kapnometrie durchgeführt?

Glasgow Coma Scale

- Es sollten die von der Therapie unbeeinflussten Werte bei Eintreffen des ersten Rettungsmittels erfasst werden.
- **Neu ab V2020:**
  - Der Herausgeber der Skala hat eine eigene deutsche Übersetzung des GCS auf seiner Homepage angeboten:
  - https://www.glasgowcomascale.org/downloads/German_v2.pdf
  - Diese Übersetzung wurde übernommen.
- Kategorienbezeichnungen bis **V2015.1**:

Glasgow Coma Scale

Augenöffnen		Verbale Antwort		Motorische Antwort	
spontan	(4)	orientiert	(5)	Aufforderung	(6)
auf Aufforderung	(3)	verwirrt	(4)	gezielt (Schmerz)	(5)
auf Schmerzreiz	(2)	inadäquat	(3)	ungezielt (Schmerz)	(4)
keine	(1)	unverständlich	(2)	Beugekrämpfe	(3)
		keine	(1)	Streckkrämpfe	(2)
				keine	(1)

- Kategorienbezeichnungen ab **V2020**:

Glasgow Coma Scale

Augenöffnen		Verbale Antwort		Motorische Antwort	
spontan	(4)	orientiert	(5)	befolgt Aufforderung	(6)
auf Geräusch	(3)	verwirrt	(4)	lokalisiert	(5)
auf Druck	(2)	Wörter	(3)	beugt normal	(4)
nicht vorhanden	(1)	Laute	(2)	beugt abnormal	(3)
nicht beurteilbar	(NT)	keine	(1)	streckt	(2)
		nicht beurteilbar	(NT)	keine	(1)
				nicht beurteilbar	(NT)

Pupillenweite

- Geänderte Variable ab V2015.
- Die Pupillengröße wird jetzt ohne Seitenangabe dokumentiert.
- Die neuen Kategorien sind: normal, Anisokorie, beidseits weit.
- Enge und mittelweite gleichmäßige Pupillengrößen sind normal.
- Erhebung vor Gabe Narkotikum!

Lichtreaktion

- Geänderte Variable ab V2015.
- Die Lichtreaktion wird jetzt ohne Seitenangabe dokumentiert.
- Die neuen Kategorien sind: spontan, verzögert, lichtstarr.
- Bei ungleicher Reaktion ist der schlechtere zu nehmen.
- Pupillenreaktion vor Gabe Narkotika erfassen!

Box Verletzungen

Neue Kategorien ab V2020:

- Hinzunahme der beiden Kategorien „geschlossen“ und „offen“ für die Verletzungsart.
- Anpassung an das DIVI-Notarzteinsatzprotokoll (Version 5.1)

Box Therapie

Neu ab V2020:
Die Reihenfolge der Maßnahmen wurde an die Reihenfolge im ATLS-Schema angepasst!

Endotracheale Intubation

- Geänderte Variable ab V2015:
  Die vorherige Variable "Intubation" wurde in "endotracheale Intubation" und "alternative Atemwegssicherung" unterteilt.
- Hier ist nur noch die endotracheale Intubation zu erfassen.

Chirurgischer Atemweg

- **Neue Variable ab V2020!**
- Hier soll jeder Atemweg erfasst werden, der chirurgisch herbeigeführt wird (Tracheotomie, Koniotomie).

Alternative Atemwegssicherung

- Geänderte Variable ab V2015:
  Die vorherige Variable "Intubation" wurde in "endotracheale Intubation" und "alternative Atemwegssicherung" unterteilt.
- Anzukreuzen, wenn nicht endotracheal intubiert, aber der Atemweg durch eine Laryxmaske oder ähnliches gesichert wurde.

HWS-Immobilisierung

- **Neue Variable ab V2020!**
- Erfasst werden soll die HWS-Immobilisation mittels Stiff-Neck, Halskragen, Head Blocks, im Sinne einer permanenten manuellen In-Line Stabilisation.

Nadeldekompression

- **Neue Variable ab V2020!**
- Gemeint ist die Nadeldekompression des Thorax zur Behebung einer Spannungskomponente bei Pneumothorax.

Thoraxdrainage

- **Geänderte Variable ab V2020!**
- Umfasst jegliche Form der Pleuraentlastung mit Ausnahme der Nadeldekompression (siehe oben).
- Hierzu zählen Bülau-, Monaldi-Drainagen, Finger-Thorakotomien (ohne Thorakotomien).

Beckengurt

- Wurde präklinisch ein Beckengurt angelegt?

Tourniquet

- **Neue Variable ab V2020!**
- Gemeint ist ein Anlegen eines Tourniquets (o.ä., z.B. Gürtel/Gummizug) zur Unterbrechung der Blutzufuhr und somit zum Stoppen der Blutung an den Extremitäten.

IO-Zugang

- **Neue Variable ab V2020!**
- Gemeint ist ein intraossär angelegter Zugang zum Infundieren von Flüssigkeit.

Herzdruckmassage

- Wurde präklinisch eine Herzdruckmassage durchgeführt?

Präklinische Thorakotomie

- **Neue Variable ab V2020!**
- Wurde präklinisch eine Thorakotomie durchgeführt (z.B. Clamshell-Thorakotomie, einseitige Thorakotomie)?

Tranexamsäure

- Wurde präklinisch Tranexamsäure (zur Blutungsstillung) verabreicht?

Katecholamine

- Wurden präklinisch Katecholamine verabreicht?

Analgosedierung

- Analgosedation umfasst Analgetika, Sedativa oder Narkotika.

Bogen B – Schockraum- und OP-Phase

Box Aufnahme

Es gilt der Zeitpunkt der Aufnahme des Patienten im Krankenhaus/in den Schockraum.
Dokumentation bei Eintritt in den Schockraum/Übergabe durch Rettungsdienst.

Neu ab V2020:
Neben dem Datum ist jetzt auch die Uhrzeit ein Pflichtfeld.

Box COVID-19

Wurde beim Patienten bei Aufnahme in das Krankenhaus ein COVID-19-Abstrich (PCR-Test) zum Nachweis des SARS-CoV-2-Virus durchgeführt? Wenn ja, welches Ergebnis brachte der Test.

Box Vitalzeichen

Es sollen die ersten gemessenen Vitalzeichen bei Übergabe des Patienten erfasst werden.

Neu ab V2020:
Die Reihenfolge der Maßnahmen wurde an die Reihenfolge im ATLS-Schema angepasst!

Herzfrequenz

- **Neu ab V2020**:
  Änderung der Bezeichnung von Puls auf Herzfrequenz.

Bereits bei Ankunft beatmet?

- War der Patient bei Einlieferung beatmet?
- Als „beatmet“ werden alle Patienten bewertet, bei denen auf Bogen A bei „Therapie“ entweder „Endotracheale Intubation“, „Chirurgischer Atemweg“ oder „Alternative Atemwegssicherung“ angegeben wurde.

FiO_2

- Für diese Variable stehen zwei Eingabe-Einheiten zur Auswahl: [%] oder [Dezimalwert]! Beispiel: 21 % entspricht 0,21.
- Bitte kontrollieren Sie die korrekte Auswahl der Einheit.
- Die gewünschte Einheit kann vom TR-Klinikadmin unter dem Menüpunkt "Festlegung Einheiten" ausgewählt werden.

PaO_2

- Für diese Variable stehen zwei Eingabe-Einheiten zur Auswahl: [mmHg] oder [kPa]!
- Bitte kontrollieren Sie die korrekte Auswahl der Einheit.
- Die gewünschte Einheit kann vom TR-Klinikadmin unter dem Menüpunkt "Festlegung Einheiten" ausgewählt werden.

Glasgow Coma Scale

- **Neu ab V2020:**
    - Der Herausgeber der Skala hat eine eigene deutsche Übersetzung des GCS auf seiner Homepage angeboten: https://www.glasgowcomascale.org/downloads/German_v2.pdf
    - Diese Übersetzung wurde übernommen.
    - Kategorienbezeichnungen ab **V2020**:

Glasgow Coma Scale

Augenöffnen		Verbale Antwort		Motorische Antwort	
spontan	(4)	orientiert	(5)	befolgt Aufforderung	(6)
auf Geräusch	(3)	verwirrt	(4)	lokalisiert	(5)
auf Druck	(2)	Wörter	(3)	beugt normal	(4)
nicht vorhanden	(1)	Laute	(2)	beugt abnormal	(3)
nicht beurteilbar	(NT)	keine	(1)	streckt	(2)
		nicht beurteilbar	(NT)	keine	(1)
				nicht beurteilbar	(NT)

- Kategorienbezeichnungen bis **V2015.1**:

Glasgow Coma Scale

Augenöffnen		Verbale Antwort		Motorische Antwort	
spontan	(4)	orientiert	(5)	Aufforderung	(6)
auf Aufforderung	(3)	verwirrt	(4)	gezielt (Schmerz)	(5)
auf Schmerzreiz	(2)	inadäquat	(3)	ungezielt (Schmerz)	(4)
keine	(1)	unverständlich	(2)	Beugekrämpfe	(3)
		keine	(1)	Streckkrämpfe	(2)
				keine	(1)

Pupillenweite

- Geänderte Variable seit 2015:
  Die Pupillengröße wird nun ohne Seitenangabe dokumentiert.
- Die neuen Kategorien sind: normal, Anisokorie, beidseits weit.
- Enge und mittelweite gleichmäßige Pupillengrößen sind normal.
- Erhebung vor Gabe Narkotikum.

Lichtreaktion

- Geänderte Variable seit V2015:
  Die Lichtreaktion wird nun ohne Seitenangabe dokumentiert.
- Die neuen Kategorien sind: spontan, verzögert, lichtstarr.
- Bei ungleicher Reaktion ist der schlechtere zu nehmen.
- Pupillenreaktion vor Gabe Narkotika erfassen!

Box Labor

Hämoglobin

- Erster HB-Wert nach Aufnahme in den Schockraum.
- Für diese Variable stehen zwei Eingabe-Einheiten zur Auswahl: [g/dl] oder [mmol/l]!
- Bitte kontrollieren Sie die korrekte Auswahl der Einheit. Die gewünschte Einheit kann vom TR-Klinikadmin unter dem Menüpunkt "Festlegung Einheiten" ausgewählt werden.

Thrombozytenzahl

- Erste Messung bei Aufnahme in den Schockraum.
- Für diese Variable stehen zwei Eingabe-Einheiten zur Auswahl: [Zellzahl/µl] oder [gpt/l]!
- Bitte kontrollieren Sie die korrekte Auswahl der Einheit. Die gewünschte Einheit kann vom TR-Klinikadmin unter dem Menüpunkt "Festlegung Einheiten" ausgewählt werden.

TPZ (Quick)

- Erster Wert nach Aufnahme in den Schockraum.

© 2020 – AUC - Akademie der Unfallchirurgie GmbH / Sektion NIS

PTT

- Erster Wert nach Aufnahme in den Schockraum.

INR

- Erster Wert nach Aufnahme in den Schockraum.

Base Excess (-/+)

- Erster Wert nach Aufnahme in den Schockraum.
- Der Base Excess kann arteriell wie venös gemessen werden.
- Bitte beachten Sie die korrekte Angabe des Vorzeichens!

Ca (ionisiert)

- Angabe des Kalzium-Titers (ionisiertes) bei der ersten Blutabnahme im Schockraum.
- Für diese Variable stehen zwei Eingabe-Einheiten zur Auswahl: [mmol/l] oder [mg/dl]!
- Bitte kontrollieren Sie die korrekte Auswahl der Einheit. Diese gewünschte Einheit kann vom TR-Klinikadmin unter dem Menüpunkt "Festlegung Einheiten" ausgewählt werden.

Ethanol (Plasma)

- Angabe des Blutalkohols, nur falls dieser gemessen wurde!
- Für diese Variable stehen zwei Eingabe-Einheiten zur Auswahl: [µmol/l] und [mg/dl]! Bitte kontrollieren Sie die korrekte Auswahl der Einheit. Die gewünschte Einheit kann vom TR-Klinikadmin unter dem Menüpunkt "Festlegung Einheiten" ausgewählt werden.

Fibrinogen

- Erster Messwert nach Aufnahme in den Schockraum.
- Für diese Variable stehen zwei Eingabe-Einheiten zur Auswahl: [µmol/l] oder [g/dl]!
- Bitte kontrollieren Sie die korrekte Auswahl der Einheit. Die gewünschte Einheit kann vom TR-Klinikadmin unter dem Menüpunkt "Festlegung Einheiten" ausgewählt werden.

Box Diagnostik

Hier ist die Durchführung der genannten Diagnostik-Maßnahmen einschließlich des Zeitpunkts des Starts der Durchführung (Beginn der Bildaufzeichnung) anzugeben.

FAST / eFAST

- **Neu ab V2020:**
- „Sono Abdomen“ umbenannt in „FAST / eFAST“
- Gemeint ist die sonographische FAST-Untersuchung des Abdomens, ggf. mit erweiterter Darstellung beider Lungenflügel (eFAST)

Freie Flüssigkeit bei FAST?

- **Neu ab V2020:**
- „Freie Flüssigkeit bei Sono Abdomen?“ umbenannt in „Freie Flüssigkeit bei FAST?“
- Wurde bei dem FAST / eFast eindeutig freie intraabdominelle Flüssigkeit festgestellt?

Selektives CT

Neu ab V2020:
Differenzierte Darstellung der selektiven CT-Diagnostik, sollte kein Ganzkörper-CT durchgeführt worden sein. Mehrfachauswahl ist möglich.

Kopf

- Selektives CT des Kopfes (cCT)

HWS

- **Neue Variable ab V2020!**
- Selektives CT der HWS (Halswirbelsäule).

Thorax mit BWS

- **Neue Variable ab V2020!**
- Selektives CT des Thorax und/oder der BWS (Brustwirbelsäule).

Abdomen/LWS/Becken

- **Neue Variable ab V2020!**
- Selektives CT des Bereiches Abdomen, LWS (Lendenwirbelsäule) und Becken.

Extremitäten

- **Neue Variable ab V2020!**
- Selektives CT der Extremitäten.

MRT

- Wurde eine Magnetresonanztomographie (MRT) im Rahmen der Erstdiagnostik durchgeführt?

ROTEM®

- Werte nur bei Verwendung eines ROTEM angeben (kein TEG, da dort andere Messwerte erhoben werden!).
- Als Zeitpunkt der Durchführung des ROTEMs ist die Zeit der Blutentnahme anzugeben.

EXTEM-CT

- Gerinnungszeit im EXTEM-Test (Aktivierung der Gerinnung durch Thromboplastin).

EXTEM-MCF

- Maximale Gerinnselfestigkeit EXTEM-Test (Aktivierung der Gerinnung durch Thromboplastin).

FIBTEM-A10

- Blockierung Thrombozyten (Beurteilung Fibrinogenspiegel und Fibrinpolymerisation).

© 2020 – AUC - Akademie der Unfallchirurgie GmbH / Sektion NIS

Nutzung Teleradiologie?

- Angabe nur bei deutschen und schweizerischen Kliniken erforderlich!
- Wurden bei diesem Fall notfallmäßig (oder auch als spätere Zweitmeinung) Bilder mit einem Teleradiologiesystem (z.B. VPN/DICOM, E-Mail/TKmed/VISUS etc.) an eine BETTEN-FUEHRENDE Abteilung (UCH, CHR, NCH, MKG/HNO etc.) versandt bzw. von diesen eine Meinung abgegeben oder - im Falle einer Zuverlegung - im eigenen Haus empfangen?
- Bei reiner Befundung durch Radiologen (Teleradiologie nach RöV) bitte NEIN ankreuzen!

Box Volumengabe

Alle Angaben beziehen sich auf den Zeitraum vom Schockraum bis zur Intensivstation, ggf. einschließlich Operation. Die präklinische Phase wird nicht berücksichtigt.

Neu ab V2020:
Die Mengenangaben zur Volumengabe können jetzt getrennt nach Schockraum- und OP-Phase eingegeben werden. Sollte die OP im Schockraum durchgeführt werden, zählt die dann gegebene Volumengabe trotzdem zur OP-Phase. Es zählt der Zeitpunkt des Hautschnitts.

Box Therapie Schockraum

Neu ab V2020:
Die Reihenfolge der Maßnahmen wurde an die Reihenfolge im ATLS-Schema angepasst!

Endotracheale Intubation

- Wurde eine endotracheale Intubation im Schockraum vorgenommen oder umintubiert?

Chirurgischer Atemweg

- **Neue Variable ab V2020!**
- Hier soll jeder Atemweg erfasst werden, der chirurgisch herbeigeführt wird (Tracheotomie, Koniotomie).

Thoraxdrainage

- Umfasst jede Form der Pleuraentlastung.

Herzdruckmassage

- Wurde im Schockraum eine Herzdruckmassage durchgeführt?

Perikardpunktion

- **Neue Variable ab V2020!**
- gemeint ist die perkutane Punktion des Perikards:
  - Mit oder ohne sonographische Kontrolle.
  - Unabhängig davon, ob ein Drain belassen wird oder nicht.

Beckengurt

- Wurde im Schockraum ein Beckengurt angelegt?
- Gemeint sind alle nicht-invasiven Tools zur Stabilisierung des Beckens.

Katecholamine (Erst-/Weitergabe)

- Wurden im Schockraum Katecholamine verabreicht?

Box Operative Notfalleingriffe bis Aufnahme ICU/IMC

Es sollen hier die angegebenen Eingriffe (nur diese elf) erfasst werden, sofern sie im SR oder im OP VOR Aufnahme ICU/IMC notfallmäßig durchgeführt wurden. Bei mehreren Eingriffen sollten die Schnittzeiten unterschiedlich angegeben werden, um eine eindeutige Reihenfolge erstellen zu können. Diese Maßnahmen/OPs bitte auch bei den „Diagnosen" erfassen. Dort auch alle hier nicht gelisteten Eingriffe und Maßnahmen erfassen.

Operative Hirndruckentlastung

- Operative Hirndruckentlastung meint Kraniotomie oder Hirndrucksonde.

Laminektomie

- Resektion von einem oder mehreren Wirbelbögen zur Entlastung des Rückenmarkes.

Thorakotomie

- Ohne Thoraxdrainage und Mini-Thorakotomie.

Laparotomie

- Chirurgisches Eröffnen der Bauchhöhle zur Durchführung eines abdominal-chirurgischen Eingriffs.

Revaskularisation

- Wiederherstellung der Durchblutung durch Gefäßanastomosen, Stenting, o.ä.

Embolisation

- (Interventionelle) Blutstillung durch Embolisation.

REBOA

- **Neue Variable ab V2020!**
- Nutzung der REBOA-Technik (Resuscitative Endovascular Balloon Occlusion of the Aorta).

Stabilisierung Becken

- Stabilisierung des Beckens durch osteosynthetische Verfahren (z.B. Fixateur, Beckenzwinge, ISG-Verschraubung, etc.).

Stabilisierung Extremitäten

- Osteosynthetische Stabilisierung der Extremitäten.

Escharotomie

- **Neue Variable ab V2020!**
- Anlage von Entlastungsschnitten der Haut bei Verbrennungen.

Dermatofasziotomie

- **Neue Variable ab V2020!**
- Gemeint ist die Dermatofasziotomie der Extremitäten zur Behebung eines Kompartmentsyndroms.

Box Hämostase-Therapie

Alle Angaben beziehen sich auf den Zeitraum vom Schockraum bis zur Aufnahme auf die Intensivstation, ggf. einschließlich Operationen. Die präklinische Phase wird nicht berücksichtigt.

Neu ab V2020:
Die Mengenangaben zur Hämostase-Therapie können jetzt getrennt nach Schockraum- und OP-Phase eingegeben werden. Sollte die OP im Schockraum durchgeführt werden, zählt die dann durchgeführte Hämostase Therapie trotzdem zur OP-Phase.

EK-Gabe: Zeitpunkt erste EK-Gabe Uhrzeit

- Zeitpunkt der ersten Gabe von EKs im Schockraum bzw. während der OP-Phase.

Frischplasma (FFP)

- Für diese Variable stehen zwei Eingabe-Einheiten zur Auswahl: [Einheiten] oder [ml]!
- Volumen-Äquivalent: 1 Einheit FFP meist 250 ml.
- Bitte kontrollieren Sie die korrekte Auswahl der Einheit! Die gewünschte Einheit kann vom TR-Klinikadmin unter dem Menüpunkt "Festlegung Einheiten" ausgewählt werden.

Thrombozytenkonzentrate

- Für diese Variable stehen zwei Eingabe-Einheiten zur Auswahl: [Einheiten] oder [ml]!
- Volumen-Äquivalent: 1 Einheit TK meist 250 ml (1 Einheit = 1 Beutel, $2{,}4 \times 10^{11}$ Thrombozyten).
- Bitte kontrollieren Sie die korrekte Auswahl der Einheit! Die gewünschte Einheit kann vom TR-Klinikadmin unter dem Menüpunkt "Festlegung Einheiten" ausgewählt werden.

Box Medikamentöse Gerinnungstherapie

Alle Angaben beziehen sich auf den Zeitraum vom Schockraum bis zur Aufnahme die Intensivstation, ggf. einschließlich Operation. Die präklinische Phase wird nicht berücksichtigt.

Tranexamsäure

- Umbenannte Variable seit V2015: Variable hieß früher "Antifibrinolytika".
- **Neu ab V2020**:
  - Angabe des Zeitpunkts der ersten Gabe.
  - Bis V2015.1 wurde der Zeitpunkt der ersten Gabe eines der genannten Medikamente abgefragt.

Fibrinogen

- Wurde zur medikamentösen Gerinnungstherapie Fibrinogen verabreicht?
- **Neu ab V2020**:
  - Angabe des Zeitpunkts der ersten Gabe.
  - Bis V2015.1 wurde der Zeitpunkt der ersten Gabe eines der genannten Medikamente abgefragt).

PPSB

- Wurde zur medikamentösen Gerinnungstherapie PPSB verabreicht?

Kalzium

- Umbenannte Variable seit V2015: Variable hieß früher "Andere hämost. Medikamente".
- Wurde zur medikamentösen Gerinnungstherapie Kalzium verabreicht?

Faktor XIII

- Wurde zur medikamentösen Gerinnungstherapie Faktor XIII verabreicht?

DOAK-Antidot

- **Neue Variable ab V2020!**
- Beispiele (derzeit in D zugelassen):
  - Idarucizumab (für Dabigatran),
  - Andexanet alfa (für Apixaban, Rivaroxaban)

Box Weiterversorgung

Weiterverlegung Uhrzeit

- Zeitpunkt Beendigung Schockraumphase bzw. akute OP-Phase.

Bogen C – Intensivstation

Box Labor / Vitalwerte

Hämoglobin

- Erster HB-Wert bei Aufnahme auf die Intensivstation.
- Für diese Variable stehen zwei Eingabe-Einheiten zur Auswahl: [g/dl] oder [mmol/l]!
- Bitte kontrollieren Sie die korrekte Auswahl der Einheit. Die gewünschte Einheit kann vom TR-Klinikadmin unter dem Menüpunkt "Festlegung Einheiten" ausgewählt werden.

Thrombozytenzahl

- Erste Messung nach Ankunft auf der Intensivstation.
- Für diese Variable stehen zwei Eingabe-Einheiten zur Auswahl: [Zellzahl/µl] oder [gpt/l]!
- Bitte kontrollieren Sie die korrekte Auswahl der Einheit. Die gewünschte Einheit kann vom TR-Klinikadmin unter dem Menüpunkt "Festlegung Einheiten" ausgewählt werden.

Base Excess (-/+)

- Erster Wert nach Aufnahme Intensivstation.
- Bitte beachten Sie die korrekte Angabe des Vorzeichens!

Ca (ionisiert)

- Neue Variable seit V2015.
- Angabe des Kalzium-Titers (ionisiertes) in [mmol/l] bei der ersten Blutentnahme auf der Intensivstation.
- Für diese Variable stehen zwei Eingabe-Einheiten zur Auswahl: [mmol/l] oder [mg/dl]!
- Bitte kontrollieren Sie die korrekte Auswahl der Einheit. Diese gewünschte Einheit kann vom TR-Klinikadmin unter dem Menüpunkt "Festlegung Einheiten" ausgewählt werden.

© 2020 – AUC - Akademie der Unfallchirurgie GmbH / Sektion NIS

Fibrinogen

- Neue Variable seit V2015.
- Erster gemessener Wert auf der Intensivstation.
- Für diese Variable stehen zwei Eingabe-Einheiten zur Auswahl: [µmol/l] oder [g/dl]!
- Bitte kontrollieren Sie die korrekte Auswahl der Einheit. Diese gewünschte Einheit kann vom TR-Klinikadmin unter dem Menüpunkt "Festlegung Einheiten" ausgewählt werden.

Box ROTEM®

EXTEM-CT

- Neue Variable seit V2015.
- Gerinnungszeit im EXTEM-Test (Aktivierung der Gerinnung durch Thromboplastin).

EXTEM-MCF

- Neue Variable seit V2015.
- Maximale Gerinnselfestigkeit im EXTEM-Test (Aktivierung der Gerinnung durch Thromboplastin).

FIBTEM-A10

- Neue Variable seit V2015.
- Gerinnselfestigkeit nach 10 Min Laufzeit: Fibrinpolymerisation nach Blockierung der Thrombozyten (Beurteilung Fibrinpolymerisation).

Box Hämostase-Therapie

Gegebene Blutprodukte in den ersten 48 Stunden NACH Intensiv-Aufnahme.

Frischplasma (FFP)

- Bitte nur die Gaben in den ersten 48 Stunden auf der Intensivstation angeben.
- Für diese Variable stehen zwei Eingabe-Einheiten zur Auswahl: [Einheiten] oder [ml]!
- Volumen-Äquivalent: 1 Einheit FFP meist 250 ml.
- Bitte kontrollieren Sie die korrekte Auswahl der Einheit. Diese gewünschte Einheit kann vom TR-Klinikadmin unter dem Menüpunkt „Festlegung Einheiten“ ausgewählt werden.

Thrombozytenkonzentrat

- **Neue Variable ab V2020!**
- Bitte nur die Gaben in den ersten 48 Stunden auf der Intensivstation angeben.
- Für diese Variable steht als Eingabe-Einheit zur Auswahl [Einheiten] oder [ml]!
- Volumen-Äquivalent: 1 Einheit TK meist 250 ml (1 Einheit = 1 Beutel, $2{,}4x10^{11}$ Thrombozyten)
- Bitte kontrollieren Sie die korrekte Auswahl der Einheit. Diese gewünschte Einheit kann vom TR-Klinikadmin unter dem Menüpunkt „Festlegung Einheiten“ ausgewählt werden.

Box Medikamentöse Gerinnungstherapie

Gegebene Medikation zur Gerinnungstherapie in den ersten 48 Stunden NACH Intensiv-Aufnahme.

Tranexamsäure

- Die Verabreichung in den ersten 48 h auf der Intensivstation ist anzugeben.

Fibrinogen

- Die Verabreichung in den ersten 48 h auf der Intensivstation ist anzugeben.

Faktor XIII

- Die Verabreichung in den ersten 48 h auf der Intensivstation ist anzugeben.

Box Aufenthaltsdauer / Beatmungsdauer

Intensivtherapie (ohne IMC)

- Gesamtaufenthaltsdauer auf Intensivstation (in Stunden oder Tagen, s.u.).
- Eine IMC gilt als Intensivstation, wenn sie 24 Stunden am Tag arztüberwacht ist.
- Bei unterbrochenen ICU-Aufenthalten bitte die kumulative Gesamtdauer angeben (Einzeltage sind zusammen zu zählen).
- Bei Angabe in Tagen bitte den ersten und letzten Tag jeweils als ganzen Tag zählen.
- Für diese Variable stehen zwei Eingabe-Einheiten zur Auswahl: [Anzahl Tage] oder [Anzahl Stunden]!
- Bitte kontrollieren Sie die korrekte Auswahl der Einheit. Diese gewünschte Einheit kann vom TR-Klinikadmin unter dem Menüpunkt "Festlegung Einheiten" ausgewählt werden.

Mechanische Beatmung

- Für diese Variable stehen zwei Eingabe-Einheiten zur Auswahl: [Anzahl Tage] oder [Anzahl Stunden]!
- Bitte kontrollieren Sie die korrekte Auswahl der Einheit. Die gewünschte Einheit kann vom TR-Klinikadmin unter dem Menüpunkt "Festlegung Einheiten" ausgewählt werden.

Box Therapie Intensivstation

ECMO (Extrakorporaler Lungensupport, Extracorporeal Membrane Oxygenation)

- Neue Variable seit 2015:
- Keine Beatmungsverfahren!

Box Organversagen

Ein OV liegt dann vor, wenn im SOFA-Score für das Organ 3 oder 4 Punkte erreicht wurden. Die jeweiligen Kriterien sind als Hilfetexte bei den einzelnen Organen hinterlegt. Angegeben wurde die Definitionen des SOFA-Scores nach Vincant et al (1996, Intensive Care Med. 22: 707-710) für Organversagen (soll an mind. 2 Tagen vorliegen).

Organversagen

- Atmung: $PaO_2/FiO_2 \leq 200$ mmHg trotz Beatmung.
- Koagulation: Thrombozyten < 50.000µl bzw. 50 gpt/l.
- Leber: Bilirubin ≥ 6,0 mg/dl
- Herz-Kreislauf: Katecholamin-Dosis: Dopamin > 5 µg/kg min ODER jegliche Adrenalin-/Noradrenalin-Gabe.
- ZNS: Glasgow Coma Scale (GCS) ≤ 9 Punkte.
- Niere: Kreatinin ≥ 3,5 mg/dl oder Ausfuhrmenge pro Tag < 500 ml/Tag.

© 2020 – AUC - Akademie der Unfallchirurgie GmbH / Sektion NIS

MOV

- Trat ein OV von mindestens zwei Organen gleichzeitig für einen längeren Zeitraum auf (mindestens zwei Tage).

Sepsis

- **Neue Definition ab V2020!**
- neue Definition:
  Sepsis ist ein lebensbedrohendes (Multi-)Organversagen, das durch eine fehlgesteuerte Immunreaktion des Wirts auf eine Infektion ausgelöst wird.
  - Berechnung über SOFA-Score:
    PaO2/FiO2, Glasgow Coma Scale, mittlerer arterieller Blutdruck (MAP) oder Einsatz von Vasopressoren, Bilirubin, Thrombozyten, Kreatinin
  - Infos zur Berechnung:
    https://www.mdcalc.com/sequential-organ-failure-assessment-sofa-score
  - Quelle: https://www.ncbi.nlm.nih.gov/pmc/articles/PMC4968574/
    (frei verfügbare Publikation)

- alte Definition (bis V2015.1):
  Sepsis war definiert als SIRS plus eine positive Blutkultur:
  - SIRS = zwei oder mehr der folgenden Kriterien erfüllt:
    - Temperatur: ≥ 38,0 °C oder ≤ 36,0 °C,
    - Puls: Tachykardie mit Herzfrequenz ≥ 90/min,
    - Atemfrequenz: ≥ 20/min oder $PaCO_2$ ≥ 4,3 kPa bzw. 33 mmHg,
    - Leukozyten: ≥ 20.000mm³ oder ≤ 4.000mm³.

Wenn Sepsis ja: Fokus?

- Neue Variable seit V2015.
- Einfachauswahl!
- Wahrscheinlichster Fokus ist zu wählen und sollte Patient mehrere septische Phasen erleben, dann die erste.
- Bei unklarem Fokus bitte „andere“ angeben.

Bogen D – Diagnosen

Es sollten alle Verletzungen erfasst werden. Dabei sind nur unmittelbare durch **das Trauma bedingte Diagnosen relevant**, nicht jedoch individuelle Traumafolgen (z.B. Schock, Kompartmentsyndrom) oder Vorerkrankungen. Die Auswahl eines **AIS-Code** ist für **jede** Diagnose verpflichtend.

Box Diagnose

Es sollen alle traumatischen Verletzungen erfasst werden.

AIS-Code

- Zur Beschreibung des Verletzungsmusters wird der Abbreviated Injury Scale (AIS-Code) verwendet, der von der "Association for the Advancement of Automotive Medicine" (AAAM) in den USA herausgegeben wird.
  - Für das TR-DGU wurde das Original-Handbuch mit rund 2000 Codes auf ca. 500 trauma-relevante Codes für die „TR-DGU - AIS-Kurzfassung AIS 2005“ reduziert.
  - Die z.Z. verwendete Version des AIS-Codes ist die Version „AIS 2005 Update 2008“ (in Verwendung seit V2009).

  - Eine Auflistung der verwendeten Codes als PDF findet sich im TraumaPortal unter: TraumaRegister DGU® > Information > Kurzfassung AIS 2005.
  - Für weitere Informationen zum AIS-Code siehe die Homepage des Herausgebers: https://www.aaam.org/abbreviated-injury-scale-ais/.
- Es muss mindestens eine Diagnose (AIS-Code) angegeben werden (Pflichtfeld).
  - Es sollen alle traumatischen Verletzungen erfasst werden.
  - Verletzungen, die in Folge anderer Verletzungen auftreten, wie z.B. das Kompartmentsyndrom, sind nicht gelistet und können nicht eingegeben werden.

Beschreibung der Verletzung / Diagnose

- Bei Auswahl einer Diagnose (AIS-Code) wird dieses Feld automatisch mit dem Beschreibungstext für den betreffenden AIS-Code befüllt.
- Dieser vergebene Text kann durch einen individuellen Text durch den Eingebenden ersetzt werden.

Diagnosestellungen nach Intensiv-Aufnahme?

- Hier kann angegeben werden, ob die Diagnosestellung für die Diagnose (AIS-Code) nach Aufnahme auf die Intensivstation erfolgte.

Box Operation

- Unter 1. und 2. sollten die beiden führenden Operationen der Diagnose aufgeführt werden (z.B. 1. Fixateur externe, 2. Plattenosteosynthese).
- Alle weiteren Operationen für diese Diagnose können mit ihrer Anzahl erfasst werden, ohne genauer klassifiziert zu werden.

OP-Maßnahme

- Geänderte Variable seit V2015.1.
- Vormals „OPS 301“ (bis 8/2019, V2015)
- Nach Auswahl einer Diagnose (AIS-Codes) steht hier eine Liste Diagnose-typischer operativer Maßnahmen zur Verfügung
- Ist die durchgeführte Maßnahme nicht aufgeführt, so sollte diese unter „Beschreibung der Operation“ genannt werden
- Für eine Liste der möglichen Maßnahmen siehe Anhang 1 (Seite *33*)

Beschreibung der Operation

- Hier können weitere Informationen zur Operation angeben werden.
- Wurde bei „OP-Maßnahmen“ „andere“ ausgewählt, bitte hier die durchgeführte Operation angeben.

Box Fraktur

Grad der Weichteilverletzung (I-V)

- Geschlossene Frakturen:
  - **Grad 0**: Keine oder unbedeutende Weichteilverletzung, indirekte Gewalteinwirkung, einfache Frakturform;
  - **Grad I**: Oberflächlich Hautabschürfung oder Quetschung Kontusion durch Fragmentdruck von innen, einfache bis mittelschwere Frakturform;
  - **Grad II**: Tiefe, verschmutzte Hautabschürfung, Kontusion durch direkte Gewalteinwirkung, drohendes Kompartmentsyndrom, mittelschwere bis schwere Frakturform;
  - **Grad III**: Ausgedehnte Hautkontusion oder Zerstörung der Muskulatur, subkutanes Decollement, manifestes Kompartmentsyndrom, Verletzung eines Hauptgefäßes.
- Offene Frakturen:
  - **Grad I**: Durchspießung der Haut, unbedeutende Verschmutzung Kontamination einfache Frakturform;
  - **Grad II**: Durchtrennung der Haut, umschriebene Haut- und Weichteilkontusion, mittelschwere Kontamination, alle Frakturformen,
  - **Grad III**: Ausgedehnte Weichteildestruktion, häufig Gefäß- und Nervenverletzung, starke Wundkontamination, ausgedehnte Knochenzertrümmerung;
  - **Grad IV**: „Subtotale“ (d. h. unvollständige) Amputationsverletzung, wobei weniger als 1/4 des Weichteilmantels intakt ist und ausgedehnte Verletzungen von Nerven und Blutgefäßen vorliegen.

Box Initiales Therapiekonzept

Angabe, ob die erste Intervention nach Damage Control-Prinzip erfolgte.

Bogen D – Abschluss

Box Entlassungs-/Todesdatum

- Bei Verlegung innerhalb 48 h oder Tod ist die Uhrzeit zusätzlich zum Datum anzugeben.

Box Entlassung/Verlegung/Tod

Bei der Angabe „Tod“

- **Geänderte Variable ab V2020!**
  (war in V2015/2015.1 „Therapiebegrenzender Patientenwille“)
- Grund für Therapiebegrenzung:
  - palliativ
    - ein mittel-/langfristiges Überleben ist aufgrund der Verletzungsschwere nach dem aktuellen Wissensstand in der Medizin nicht möglich.
  - mutmaßlicher Patientenwille
    - Dieser Wille ist bei Fehlen einer Patientenverfügung aus früheren Äußerung und Wertevorstellungen des Patienten ermittelbar, z.B. im Rahmen von interdisziplinären Fall- und/oder Ethikkonferenzen zusammen mit den Angehörigen/Betreuer des Patienten.
  - schriftlich bekundeter Patientenwille
    - Vorliegen einer individuellen schriftlichen Patientenverfügung oder ähnlichen Dokumenten, in denen der Wille des Patienten klar formuliert ist.

Box Zustand bei Entlassung/Verlegung

- **Geänderte und umbenannte Variable ab V2020!**
- Die Variable war für V2015/2015.1 von „Glasgow Outcome Scale (GOS)“ in „Outcome“ umbenannt worden, da der GOS per Definition auf SHT-Patienten beschränkt ist. Die Umbenennung sollte deutlich machen, dass die Variable allgemein für alle Patienten gilt.
- Erneute Umbenennung für V2020 in „Zustand bei Entlassung/Verlegung“, um herauszustellen das der Zustand im Moment der Entlassung bzw. Verlegung gemeint ist.
- Zur Vereinfachung der Eingabe wurde die Kategorie „tot“ aus dem Erhebungsbogen herausgenommen (bei der Angabe „tot“ bei „Entlassung/Verlegung/Tod“ wird diese Angabe automatisch bei der Speicherung des Falles für „Zustand bei Entlassung/Verlegung“ in die Datenbank übernommen).
- Für die einzelnen Kategorien gelten folgende Definitionen:
  - **gut erholt**: Gute Erholung.
  - **mäßig behindert**: Mäßige Behinderung (behindert, aber unabhängig).
  - **schwer behindert**: Schwere Behinderung (bei Bewusstsein, aber behindert und auf Hilfe angewiesen).
  - **nicht ansprechbar:** Persistierender vegetativer Zustand.
  -

Box Todesursache

Todesursache

- Todesursächliches Organsystem, das traumaassoziiert als Ursache für den Tod gilt.
- Für die einzelnen Kategorien gelten folgende Definitionen:
  - **SHT:** Das Schädel-Hirn-Trauma führt unmittelbar zum Tode oder die Therapie wurde auf Grund der Schwere des Schädel-Hirn-Traumas eingestellt.
  - **Hämorrhagie:** Versterben auf Grund einer traumaassoziierten Blutung. Multiorganversagen auf Grund eines prologierten Schocks ist als „Organversagen“ einzustufen. Blutungen, die unabhängig vom Trauma auftreten, z. B. gastrointestinale Blutung, sind als „Sonstige“ Todesursache einzustufen.
  - **Organversagen:** Versterben auf Grund eines progredienten Organversagens (Multiorganversagen). Dies kann durch eine Sepsis, in Folge eines Blutungsschocks o. a. begründet sein. Nicht gemeint sind ein akuter Herzinfarkt oder eine Lungenembolie. Hier ist „Sonstige“ zu wählen.
  - **Sonstige**: Versterben nicht auf Grund von Schädel-Hirn-Trauma, Hämorrhagie oder Organversagen. Hier kämen z. B. Lungenembolie, Herzinfarkt, Spannungspneumothorax, hoher Querschnitt o. ä. in Betracht.

Anhang 1

OP-Gruppen zur Angabe der durchgeführten Operationen

Bis 2018 erfasste das TraumaRegister DGU® zu den Operationen den jeweiligen OPS-Code. Mitte 2019 wurde dieses Feld aus dem Datensatz herausgenommen und gegen eine Liste möglicher OP-Maßnahmen für die jeweilige Diagnose ersetzt.

Dafür wurden OP-Maßnahmen, die bei einem bestimmten Verletzungsmuster durchgeführt werden können, in einer gemeinsamen „OP-Gruppe" zusammengefasst. Jeder Diagnose (AIS-Code) wurde anschließend eine dieser „OP-Gruppen" zugeordnet. In den Eingabemasken können bei „OP-Maßnahmen" nach der Auswahl einer Diagnose (AIS-Code) dann bis zu zwei dieser Maßnahmen ausgewählt werden (siehe auch *Bogen D – Diagnosen* auf Seite *29*).

Einige OP-Maßnahmen kommen in mehreren OP-Gruppen vor.

OP-Gruppe 0

(keine OP-Maßnahme durchgeführt)

OP-Gruppe 1

(Verletzungen des Gehirns, einschließlich penetrierender Verletzungen bei Hirn-Beteiligung)

Entdeckelung
Hämatomentlastung
Parenchymdrucksonde
Ventrikeldrainage

OP-Gruppe 2

(Schädelbasisfraktur, Nase, Zähne)

Cerclage / Zuggurtung / Draht
Lappenplastik (Rotation, Fasziocutan, Muskel)
Plattenosteosynthese
Rekonstruktion Frontobasis
Replantation
Reposition, geschlossen
Schraubenosteosynthese

OP-Gruppe 3

(Penetrierende Verletzungen (ohne Hirnbeteiligung), Weichteilverletzungen, Elektrizitätsunfall)

Chirurgische Blutungskontrolle
Escharotomie
Fremdkörperentfernung
Kompartmentspaltung
Lappenplastik (Rotation, Fasziocutan, Muskel)
Naht / Rekonstruktion inkl. Debridement
Temporäre Deckung inkl. Debridement (VAC, Epigard, ...)

OP-Gruppe 4

(Gefäßverletzungen)

Ballonokklusion
Bypass
Chirurgischer Verschluss (Ligatur, ...)
Interponat (inkl. Prothese)
Interventioneller Verschluss (Coiling, ...)
Rekonstruktion (Naht / Patch)
Stent

OP-Gruppe 5

(Nerven- und Rückenmarksverletzungen)

Interponat / Tubing
Laminektomie
Naht (Koaptation)
Neurolyse / Dekompression

OP-Gruppe 6

(Retroperitonealhämatom, innere Organe; Verletzungen an Hals-Organen, Mundhöhle und Ohr)

Ballonokklusion
Blindverschluss
Chirurgische Blutungskontrolle
Drainage / Entlastung
Exploration
Externe Organausleitung (Enterostoma/Fistel/AP)
Interventionelle Blutungskontrolle
Naht / Rekonstruktion (prim./sek.)
Resektion
Stent
Teilresektion
Tracheostoma / Koniotomie

OP-Gruppe 7

(Knöcherne Verletzungen am Thorax und den Extremitäten)

Amputation / Stumpfversorgung
Arthrodese
Cerclage / Zuggurtung / Draht
Externe Stabilisierung (FixEx, ...)
Intramedulläre Schienung
Lappenplastik (Rotation, Fasziocutan, Muskel)
Nagelosteosynthese
Plattenosteosynthese
Prothese / Gelenk(teil)ersatz
Replantation
Reposition, geschlossen
Resektionsarthroplastik
Schraubenosteosynthese

© 2020 – AUC - Akademie der Unfallchirurgie GmbH / Sektion NIS

OP-Gruppe 8

(Becken-Verletzungen)

Amputation / Stumpfversorgung
Arthrodese
Ballonokklusion
Chirurgische Blutungskontrolle
Externe Stabilisierung (FixEx, ...)
Interventionelle Blutungskontrolle
Lappenplastik (Rotation, Fasziocutan, Muskel)
Plattenosteosynthese
Prothese / Gelenk(teil)ersatz
Replantation
Reposition, geschlossen
Resektionsarthroplastik
Schraubenosteosynthese

OP-Gruppe 9

(Knöcherne / ligamentäre Wirbelsäule (ohne Rückenmarksverletzung); Bandscheiben)

Dorsale Stabilisierung
Halo-Fixateur
Laminektomie / Dekompression
Ventrale Stabilisierung

© 2020 – AUC - Akademie der Unfallchirurgie GmbH / Sektion NIS
